# Two New Loci for Body-Weight Regulation Identified in a Joint Analysis of Genome-Wide Association Studies for Early-Onset Extreme Obesity in French and German Study Groups

**DOI:** 10.1371/journal.pgen.1000916

**Published:** 2010-04-22

**Authors:** André Scherag, Christian Dina, Anke Hinney, Vincent Vatin, Susann Scherag, Carla I. G. Vogel, Timo D. Müller, Harald Grallert, H.-Erich Wichmann, Beverley Balkau, Barbara Heude, Marjo-Riitta Jarvelin, Anna-Liisa Hartikainen, Claire Levy-Marchal, Jacques Weill, Jérôme Delplanque, Antje Körner, Wieland Kiess, Peter Kovacs, Nigel W. Rayner, Inga Prokopenko, Mark I. McCarthy, Helmut Schäfer, Ivonne Jarick, Heiner Boeing, Eva Fisher, Thomas Reinehr, Joachim Heinrich, Peter Rzehak, Dietrich Berdel, Michael Borte, Heike Biebermann, Heiko Krude, Dieter Rosskopf, Christian Rimmbach, Winfried Rief, Tobias Fromme, Martin Klingenspor, Annette Schürmann, Nadja Schulz, Markus M. Nöthen, Thomas W. Mühleisen, Raimund Erbel, Karl-Heinz Jöckel, Susanne Moebus, Tanja Boes, Thomas Illig, Philippe Froguel, Johannes Hebebrand, David Meyre

**Affiliations:** 1 Institute for Medical Informatics, Biometry and Epidemiology, University of Duisburg-Essen, Essen, Germany; 2 Department of Child and Adolescent Psychiatry, University of Duisburg-Essen, Essen, Germany; 3 Centre National de la Recherche Scientifique (CNRS) 8090-Institute of Biology, Pasteur Institute, Lille, France; 4 Department of Psychiatry, University of Cincinnati Genome Research Institute, Cincinnati, Ohio, United States of America; 5 Helmholtz Zentrum München, German Research Center for Environmental Health, Institute of Epidemiology, Munich-Neuherberg, Germany; 6 Institute of Medical Data Management, Biometrics, and Epidemiology, Ludwig-Maximilians University Munich, Munich, Germany; 7 INSERM, CESP Centre for Research in Epidemiology and Population Health, U1018, Epidemiology of Diabetes, Obesity and Chronic Kidney Disease over the Lifecourse, Université Paris Sud 11, UMRS 1018, Villejuif, France; 8 Department of Epidemiology and Public Health, Imperial College London, London, United Kingdom; 9 Institute of Health Sciences, Department of Child and Adolescent Health, National Public Health Institute, Biocenter Oulu, University of Oulu, Oulu, Finland; 10 Department of Clinical Sciences/Obstetrics and Gynecology, University of Oulu, Oulu, Finland; 11 INSERM, U690, Université Paris Diderot, Paris, France; 12 Pediatric Endocrine Unit, Jeanne de Flandre Hospital, Lille, France; 13 University Hospital for Children and Adolescents, University of Leipzig, Leipzig, Germany; 14 Department of Internal Medicine III, Interdisciplinary Centre for Clinical Research, University of Leipzig, Leipzig, Germany; 15 Oxford Centre for Diabetes, Endocrinology and Metabolism, University of Oxford, Churchill Hospital, Oxford, United Kingdom; 16 The Wellcome Trust Centre for Human Genetics, University of Oxford, Oxford, United Kingdom; 17 Institute of Medical Biometry and Epidemiology, Philipps-University of Marburg, Marburg, Germany; 18 Department of Epidemiology, German Institute of Human Nutrition Potsdam-Rehbrücke, Nuthetal, Germany; 19 Institute for Paediatric Nutrition Medicine, Vestische Hospital for Children and Adolescents, University of Witten/Herdecke, Datteln, Germany; 20 Department of Paediatrics, Marien-Hospital Wesel, Wesel, Germany; 21 Children's Hospital, Municipal Hospital “St Georg”, Leipzig, Germany; 22 Department of Pediatrics, University of Leipzig, Leipzig, Germany; 23 Institute of Experimental Pediatric Endocrinology, Charité-Universitätsmedizin Berlin, Berlin, Germany; 24 Institute for Pharmacology, Ernst-Moritz-Arndt University, Greifswald, Germany; 25 Department of Clinical Psychology and Psychotherapy, Faculty of Psychology, University of Marburg, Germany; 26 Molecular Nutritional Medicine, Technische Universität München, Else Kröner-Fresenius Center, Freising-Weihenstephan, Germany; 27 Department of Pharmacology, German Institute of Human Nutrition Potsdam-Rehbruecke, Nuthetal, Germany; 28 Institute of Human Genetics, University of Bonn, Bonn, Germany; 29 Department of Genomics, Life and Brain Center, University of Bonn, Bonn, Germany; 30 Department of Cardiology, University of Duisburg-Essen, Essen, Germany; 31 Department of Genomic Medicine, Imperial College London, Hammersmith Hospital, London, United Kingdom; University of Geneva Medical School, Switzerland

## Abstract

Meta-analyses of population-based genome-wide association studies (GWAS) in adults have recently led to the detection of new genetic loci for obesity. Here we aimed to discover additional obesity loci in extremely obese children and adolescents. We also investigated if these results generalize by estimating the effects of these obesity loci in adults and in population-based samples including both children and adults. We jointly analysed two GWAS of 2,258 individuals and followed-up the best, according to lowest p-values, 44 single nucleotide polymorphisms (SNP) from 21 genomic regions in 3,141 individuals. After this DISCOVERY step, we explored if the findings derived from the extremely obese children and adolescents (10 SNPs from 5 genomic regions) generalized to (i) the population level and (ii) to adults by genotyping another 31,182 individuals (GENERALIZATION step). Apart from previously identified *FTO, MC4R*, and *TMEM18*, we detected two new loci for obesity: one in *SDCCAG8* (serologically defined colon cancer antigen 8 gene; p = 1.85×10^−8^ in the DISCOVERY step) and one between *TNKS* (tankyrase, TRF1-interacting ankyrin-related ADP-ribose polymerase gene) and *MSRA* (methionine sulfoxide reductase A gene; p = 4.84×10^−7^), the latter finding being limited to children and adolescents as demonstrated in the GENERALIZATION step. The odds ratios for early-onset obesity were estimated at ∼1.10 per risk allele for both loci. Interestingly, the *TNKS/MSRA* locus has recently been found to be associated with adult waist circumference. In summary, we have completed a meta-analysis of two GWAS which both focus on extremely obese children and adolescents and replicated our findings in a large followed-up data set. We observed that genetic variants in or near *FTO, MC4R, TMEM18, SDCCAG8*, and *TNKS*/*MSRA* were robustly associated with early-onset obesity. We conclude that the currently known major common variants related to obesity overlap to a substantial degree between children and adults.

## Author Summary

Genome-wide association studies (GWAS) have successfully contributed to the detection of genetic variants involved in body-weight regulation. We jointly analysed two GWAS for early-onset extreme obesity in 2,258 individuals of European origin and followed-up the findings in 3,141 individuals. Evidence for association of markers in two new genetic loci was shown (*SDCCAG8* on chromosome 1q43–q44 and between *TNKS/MSRA* on chromosome 8p23.1). We also re-identified variants in or near *FTO*, *MC4R*, and *TMEM18* to be associated with extreme obesity. In addition, we assessed the effect of the markers in 31,182 obese, lean, normal weight, and unselected individuals from population-based samples and showed that the variants near *FTO, MC4R, TMEM18*, and *SDCCAG8* were consistently associated with obesity. For variants of *TNKS/MSRA*, the obesity association was limited to children and adolescents. In summary, we detected two new obesity loci and confirmed that the currently known major common variants related to obesity overlap to a substantial degree between children and adults.

## Introduction

Recent genome-wide association studies (GWAS) conducted in adult population-based samples assessed for body mass index (BMI) or in case-control designs for extreme obesity led to the discovery of genetic loci relevant for body weight regulation. The first genetic loci were detected via variants in intron 1 of the *FTO* (fat mass and obesity associated gene; e.g., [1]–[4]) and variants approx. 200 kb downstream of *MC4R* (melanocortin 4 receptor gene; [5]–[8]) reported by the GIANT (Genetic Investigation of ANthropometric Traits) consortium. This consortium subsequently detected six additional genetic loci relevant for BMI in a meta-analysis of 15 GWAS based on 32,387 probands and large confirmation samples (>58,000 individuals; with single nucleotide polymorphisms (SNP) in or near *TMEM18*, transmembrane protein 18 gene; *KCTD15*, potassium channel tetramerization domain containing 15 gene; *GNPDA2*, glucosamine-6-phosphate deaminase 2 gene; *SH2B1*, SH2B adapter protein 1 gene; *MTCH2*, mitochondrial carrier homologue 2 gene; *NEGR1*, neuronal growth regulator 1 gene). In parallel, a combined analysis of 34,416 individuals from Iceland, the Netherlands, North America (European and African descent) and Scandinavia revealed 11 regions of genome-wide significance at ≤1.6×10^−7^ (in or near *FTO*; *MC4R*; *TMEM18*; *KCTD15*; *SH2B1; NEGR1; SEC16B*, SEC16 homologue B gene; *ETV5*, ets variant gene 5; *BDNF*, brain-derived neurotrophic factor gene and two gene rich loci on chromosome 6p21.33 and 12q13.13 with the closest genes *AIF1*, allograft inflammatory factor 1 gene, and *BCDIN3D*, BCDIN3 domain containing gene, respectively). Finally, shifting to the analysis of extremely obese subjects, Meyre et al. [9] analyzed GWAS data from 1,380 Europeans with early-onset and morbid adult obesity and 1,416 age-matched normal-weight controls and reported three new risk loci in *NPC1* (Niemann-Pick disease, type C1 gene), near *MAF* (v-maf musculoaponeurotic fibrosarcoma oncogene homolog gene) and *PTER* (phosphotriesterase related gene), which were followed-up in 14,186 European subjects. Altogether, 16 genetic loci relevant for body weight regulation have been identified by these three GWAS approaches [10]–[12].

**Figure 1:**
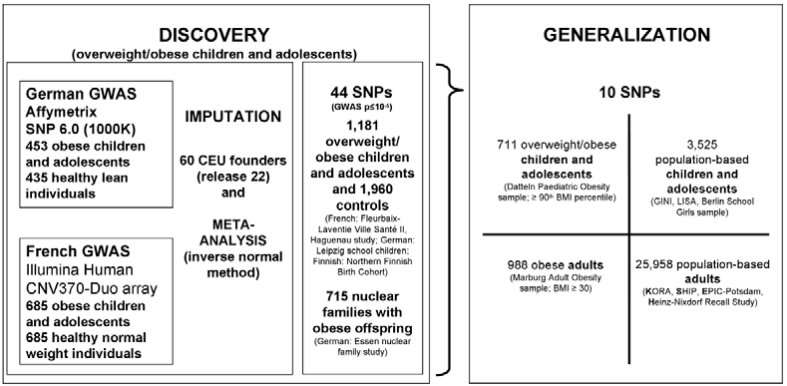
Study design to discover consistently associated genetic loci for (early-onset) obesity. In the DISCOVERY step we jointly analysed two GWAS focussing on extremely obese children and adolescents. Markers with the smallest p-values of the GWAS were validated in independent case-control and nuclear family samples again with a focus on overweight/obese children and adolescents. Afterwards, in the GENERALIZATION step, we extended the focus in two dimensions—(i) from the extremes to the population level and (ii) from children and adolescents to adults. Note that we used controls selected from the population-based samples for the cases-control comparison with obese individuals for the GENERALIZATION (BMI quartile < median for children & BMI <25 kg/m^2^ for adults).

While meta-analytic combinations of multiple GWAS were highly successful in population-based samples, no such approach has up to now been applied to case-control designs for obesity. Here we combined GWAS based on two samples that were specifically ascertained for the analysis of paediatric extreme obesity [3], [9]. We aimed to identify genetic loci that are relevant for early onset extreme obesity and to determine effect sizes of such loci for obesity in adults and in population-based samples including both children and adults (see Figure 1 for the general design of the study).

In particular, our study design was based on two steps to enable hypothesis-free SNP identification and confirmation. In the DISCOVERY step, we screened 2,239,392 genotyped or imputed SNPs and tested 1,596,878 SNPs (after quality control) for association in a combined French and German sample of 1,138 extremely obese children and adolescents and 1,120 normal- or underweight controls as based on a minor allele frequency above 1%. Next, we (*de novo*) genotyped all SNPs with strong evidence for an association to obesity (according to p-value ranking; for details see “Materials and Methods” and Text S1) in independent samples of 1,181 obese children and adolescents and 1,960 normal- or underweight controls and in up to 715 nuclear families with at least one extremely obese offspring. In the GENERALIZATION step, we extended the focus of our study in two dimensions - (i) from children and adolescents to adults and (ii) from (extreme) obesity to the population level (in sum we (*de novo*) genotyped 31,182 individuals in the GENERALIZATION step).

In addition to our hypothesis-free step-wise design, we aimed to re-confirm the associations of the recently reported GWAS-based genetic loci for body weight regulation [9], [13], [14] in our paediatric extreme obesity GWAS meta-analysis.

## Results

**Figure 2:**
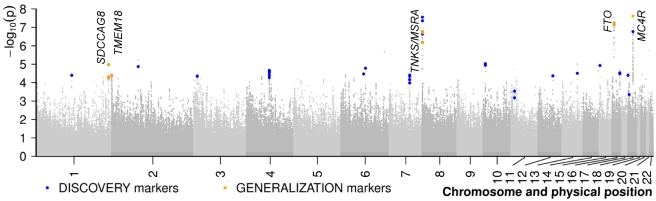
Results of the meta-analysis of two genome-wide association studies for early-onset extreme obesity. SNPs are plotted on the x-axis according to their position on each chromosome (HapMap, release 22) against the association signal on the y-axis (shown as -log_10_ of the two-sided (deflated/adjusted) p-value). SNPs genotyped in the independent samples of the DISCOVERY step are shown as blue circles (some of them are proxy SNPs of the best signals). SNPs followed-up in the GENERALIZATION step as well are shown as orange squares. For details on the marker selection see Text S1.

In our GWAS meta-analysis based on the German and French study groups encompassing both young obese cases and normal weight or lean controls we discovered three SNPs with genome-wide significance (Table 1 and Figure 2, Figure S1) even when applying the conservative Bonferroni correction at α_BF_≈3.1×10^−8^ for all 1,596,878 SNPs. While two markers are located in the previously reported *FTO* (intron 1; rs1421085; p = 2.99×10^−8^) and downstream of *MC4R* (rs17700144; p = 2.40×10^−8^), rs473034 indicates a new genetic locus for early onset extreme obesity located on chromosome 8p23.1 (p = 2.77×10^−8^) with the closest genes *TNKS* (tankyrase, TRF1-interacting, ankyrin-related ADP-ribose polymerase gene; ∼135 kb upstream of rs473034) and *MSRA* (methionine sulfoxide reductase A gene; ∼178 kb downstream of rs473034). In addition to the three genome-wide significant regions, the GWAS data revealed 18 genomic regions of interest which were defined by (i) two-sided p-values of a lead SNPs ≤10^−5^ and (ii) more than a single SNP within a locus (lead SNP ±500 kb) showing evidence for association as defined via a p-value rank <1,500 (roughly corresponding to p≤5×10^−4^; for details see Text S1).

**Table 1:** DISCOVERY and GENERALIZATION.

		DISCOVERY					GENERALIZATION			
		children and adolescents (n = 1,138 extreme obese cases; n = 1,120 lean or normal weight controls)		children and adolescents (n = 1,181 obese and overweight cases; n = 1,960 normal weight controls^c^; 715 nuclear families with obese offspring)			children and adolescents (n = 711 obese cases; n = 1,803 normal weight controls^c^)		adults (n = 988 obese cases; n = 4,117 normal weight controls^c^)	
chromosomal region^a^ (suggested gene)	SNP (obesity risk effect allele^b^)	odds ratio for effect allele in French/German GWAS	combined p-value	combined odds ratio for effect allele (95% CI)	combined p-value	combined p-value^d^	odds ratio for effect allele (95% CI)	p-value	odds ratio for effect allele (95% CI)	p-value
1q43–q44 (*SDCCAG8*)	rs10926984 (T)	1.39/1.52	4.86×10^−5^	1.16 (1.03;1.33)	0.02	3.94×10^−6^	1.12 (0.94;1.33)	0.20	1.19 (1.03;1.39)	0.03
	rs12145833 (T)	1.41/1.52	1.05×10^−5^	1.19 (1.05;1.37)	0.01	4.84×10^−7^	1.15 (0.96;1.37)	0.12	1.18 (1.02;1.35)	0.03
	rs2783963 (C)	1.41/1.41	5.63×10^−5^	1.15 (1.01;1.30)	0.04	8.68×10^−6^	1.10 (0.92;1.32)	0.28	1.18 (1.01;1.35)	0.03
2p25.3 (*TMEM18*)	rs11127485 (T)	1.32/1.64	4.18×10^−5^	1.23 (1.08;1.41)	7.56×10^−4^	1.64×10^−7^	1.35 (1.14;1.61)	5.87×10^−4^	1.45 (1.25;1.67)	3.48×10^−7^
8p23.1 (*TNKS/MSRA*)^e^	rs17150703 (A)	1.78/1.75	1.69×10^−7^	1.18 (1.03;1.35)	0.02	1.85×10^−8^	1.06 (0.86;1.30)	0.59	1.00 (0.85;1.18)	0.99
	rs13278851 (A)	1.78/1.74	1.92×10^−7^	1.29 (1.04;1.61)	0.02	2.09×10^−8^	1.05 (0.86;1.29)	0.61	0.98 (0.83;1.15)	0.77
	rs516175 (T)	1.78/1.59	6.54×10^−7^	1.16 (1.02;1.32)	0.03	9.88×10^−8^	1.12 (0.93;1.35)	0.25	0.97 (0.82;1.13)	0.66
16q12.2 (*FTO*)^e^	rs1558902 (A)	1.33/1.54	5.74×10^−8^	1.37 (1.26;1.50)	7.41×10^−13^	5.00×10^−19^	1.35 (1.19;1.52)	1.78×10^−6^	1.45 (1.31;1.60)	2.12×10^−13^
	rs9935401 (A)	1.35/1.53	6.92×10^−8^	1.20 (1.05;1.38)	0.01	4.06×10^−9^	1.35 (1.20;1.53)	1.70×10^−6^	1.35 (1.22;1.49)	4.58×10^−9^
18q21.32 (*MC4R*)	rs17700144 (A)	1.48/1.50	2.40×10^−8^	*1.22* f *(1.09;1.37)*	*3.88*×*10^−4^*	6.47×10^−11^	1.44 (1.25;1.66)	6.85×10^−7^	1.14 (1.01;1.28)	0.03

Evidence for qualitative (cases versus controls) associations for 5 loci (10 SNPs) under a log-additive genetic model. Effect sizes as point estimators and 95% confidence intervals (95% CI), p-values (two-sided) and combined p-values are presented; all samples are described in detail in Text S1.

**^a^**position and stranding according to dbSNP BUILD 129; Map to Genome Build 36.3;

**^b^**(obesity) effect risk alleles as derived from the paediatric extreme obesity GWAS meta-analysis;

**^c^**from all population-based children and adolescents samples (GINI/LISA and Berlin School Girls sample with a BMI quantile < age- and gender-matched median) or adults (KORA with BMI <25 kg/m^2^); all exact two-sided p-values of HWE test >0.01;

**^d^**combined p-values by Fischer combination rule;

**^e^**rs473034 (*TNKS/MSRA*) and rs1421085 (*FTO*) had genome-wide significant p-values of 2.77×10^−8^ and 2.99×10^−8^ in the paediatric extreme obesity GWAS meta-analysis but proxies were chosen for the follow-up;

**^f^**results for rs17700144 (*MC4R*) are based on the proxy marker rs10871777.

As part of our DISCOVERY step, we subsequently (*de novo*) genotyped 44 SNPs representing these 21 genomic regions of interest in independent 1,181 obese children and adolescents and 1,960 normal- or underweight controls and in up to 715 nuclear families with at least one extremely obese offspring (Table 1; Table S3). For 5 out of the 21 regions the association was directionally consistent (i.e. we observed the same obesity risk effect allele as in our GWAS meta-analysis) and the minimum combined p-value for each region across the samples was p≤5×10^−4^ (Table 1; for details see Text S1). These 5 genomic regions included three known loci on chromosome 2p25.3 (*TMEM18)*, 16q12.2 (*FTO*), 18q21.32 (3′ of *MC4R*) as well as two new loci on chromosome 1q43-q44 and on chromosome 8p23.1 (Figure 2, Figure S2). The SNPs of the first new locus on chromosome 1q43-q44 are located within introns of the *SDCCAG8* (serologically defined colon cancer antigen 8 gene) whereas the second new locus on chromosome 8p23.1 between the *TNKS* and *MSRA* had already showed evidence for an association at the genome-wide level in the initial paediatric extreme obesity GWAS meta-analysis.

Based on these results, we extended the focus of our study in two dimensions - from children and adolescents to adults and from the extremes to the population level - looking for GENERALIZATION of the replicated 5 regions represented by 10 SNPs (Table 1). Comparing children and adolescents to adults using case-control designs with overweight and obese cases vs. normal weight controls revealed directionally consistent (see above) findings for the variants of *FTO*, *TMEM18* and the novel *SDCCAG8* (Table 1). Similarly the odds ratios for the respective obesity risk effect alleles did not vary strongly by group (children and adolescents vs. adults) with point estimates ranging between 1.35–1.45 (*FTO*), 1.35–1.45 (*TMEM18*) and 1.10–1.19 (*SDCCAG8*). For the SNPs related to *MC4R* and the new *TNKS/MSRA* locus, however, we observed age dependent differences: For *MC4R*, we confirmed the findings by Loos and co-workers [6] by finding a stronger effect size estimator in children and adolescents as compared to adults (1.44 vs. 1.14 for rs17700144 of *MC4R*; p = 9.39×10^−3^ for the interaction of genotype and group). For *TNKS/MSRA*, we found an effect in children and adolescents but no effect in adults (e.g., 1.12 vs. 0.97 for rs516175). These differences in obesity risk effects between children and adolescents as compared to adults, however, were not due to large differences in allele frequencies as based on the population-based samples with a maximum difference of 0.82% for rs11127485 of *TMEM18*. We then compared (extreme) obesity assessed in case-control designs to the analyses of quantitative BMI data derived from population-based samples in the GENERALIZATION step (3,525 children and adolescents and 25,958 adults of European origin; Table 1, Table 2). BMI analyses revealed that the two SNPs in *FTO* and *TMEM18* would have also been detectable using population-based samples of the given sizes from children/adolescents and adults (p-values 7.87×10^−4^ and 9.99×10^−16^ for *FTO* and 0.01 and 9.97×10^−12^ for *TMEM18* with the values in the adults being even significant at a stringent genome-wide significance level of α = 5×10^−8^). The *MC4R* SNP, however, would have been harder to detect (p-values of 0.02 for children and adolescents and 1.10×10^−4^ for adults); detection of the two new loci *SDCCAG8* and *TNKS/MSRA* would have been impossible (Table 2).

**Table 2:** GENERALIZATION.

		GENERALIZATION					
		children and adolescents (n = 3,525)		adults (n = 25,958)			
chromosomal region^a^ (suggested gene)	SNP (obesity risk effect allele^b^)	BMI-SDS^d^ estimator (beta) for effect allele (95% CI)	p-value	BMI estimator (beta) for effect allele (95% CI)	p-value	combined BMI estimator (beta) for effect allele (95% CI)^e^	combined p-value^e^
1q43–q44 (*SDCCAG8*)	rs10926984 (T)	0.016 (−0.043;0.076)	0.59	K 0.04 (−0.11;0.19)	0.57	0.02 (−0.10; 0.13)	0.79
				E 0.01 (−0.22;0.25)	0.91		
				H −0.05 (−0.31;0.21)	0.73		
	rs12145833 (T)	−0.008 (−0.067;0.050)	0.78	K 0.07 (−0.08;0.22)	0.39	0.05 (−0.05;0.16)	0.33
				S 0.18 (−0.09;0.44)	0.20		
				E 0.01 (−0.22;0.25)	0.92		
				H −0.07 (−0.33;0.19)	0.60		
	rs2783963 (C)	0.006 (−0.054;0.066)	0.85	K 0.03 (−0.12;0.18)	0.70	0.02 (−0.10;0.13)	0.79
				E 0.01 (−0.23;0.25)	0.94		
				H −0.02 (−0.28;0.24)	0.90		
2p25.3 (*TMEM18*)	rs11127485 (T)	0.079 (−0.021;0.136)	0.01	K 0.37 (0.22;0.52)	9.26×10^−7^	0.35 (0.25;0.45)	9.97×10^−12^
				S 0.46 (0.20;0.72)	0.001		
				E 0.19 (−0.04;0.42)	0.11		
				H 0.38 (0.14;0.63)	0.002		
8p23.1 (*TNKS/MSRA*)	rs17150703 (A)	−0.028 (−0.103;0.047)	0.46	K −0.12 (−0.31;−0.06)	0.19	−0.10 (−0.23;0.03)	0.12
				S −0.16 (−0.49;0.18)	0.37		
				E 0.01 (−0.28;0.31)	0.93		
				H −0.13 (−0.44;0.19)	0.43		
	rs13278851 (A)	−0.022 (−0.096;0.052)	0.56	K −0.13 (−0.31;0.06)	0.18	−0.10 (−0.24;0.04)	0.15
				E 0.00 (−0.30;0.30)	0.99		
				H −0.13 (−0.44;0.19)	0.44		
	rs516175 (T)	−0.004 (−0.073;0.064)	0.90	K −0.03 (−0.20;0.15)	0.76	−0.04 (−0.16;0.08)	0.49
				S −0.13 (−0.45;0.19)	0.42		
				E 0.00 (−0.29;0.26)	0.99		
				H −0.05 (−0.35;0.26)	0.77		
16q12.2 (*FTO*)	rs1558902 (A)	0.074 (0.031;0.116)	7.87×10^−4^	K 0.29 (0.18;0.40)	1.34×10^−7^	0.31 (0.24;0.39)	9.99×10^−16^
				S 0.19 (−0.01;0.39)	0.07		
				E 0.44 (0.27;0.61)	4.69×10^−7^		
				H 0.34 (0.14;0.53)	7.76×10^−4^		
	rs9935401 (A)	0.074 (0.030;0.117)	9.04×10^−4^	K 0.29 (0.18;0.40)	3.35×10^−7^	0.30 (0.23;0.38)	7.99×10^−15^
				S 0.17 (−0.02;0.37)	0.09		
				E 0.44 (0.26;0.60)	1.36×10^−6^		
				H 0.33 (0.13;0.53)	0.001		
18q21.32 (*MC4R*)	rs17700144 (A)	0.064 (0.011;0.116)	0.02	K 0.10 (−0.03;0.23)	0.13	0.17 (0.08;0.26)	1.10×10^−4^
				S 0.28 (0.05;0.51)	0.02		
				E 0.18 (−0.02;0.39)	0.08		
				H 0.27 (0.04;0.50)	0.02		

Evidence for quantitative associations (BMI or standard deviation score of BMI (BMI-SDS)) assessed cross-sectionally for 5 loci (10 SNPs) under an additive genetic model. Effect sizes as point estimators and 95% confidence intervals (95% CI), p-values (two-sided) and combined effect estimators and p-values are presented for the adults with abbreviations for the samples: **K**ORA, **S**HIP^c^, **E**PIC-Potsdam, **H**einz-Nixdorf Recall Study; all samples are described in detail in Text S1.

**^a^**position and stranding according to dbSNP BUILD 129; Map to Genome Build 36.3;

**^b^**(obesity) effect risk alleles as derived from the paediatric extreme obesity GWAS meta-analysis;

**^c^**results for SHIP are based on *in silico* GWAS data–proxy markers (*FTO*: rs8050136 for rs9935401, rs1421085 for rs1558902; *MC4R*: rs476828 for rs17700144); one marker for each region was regarded as sufficient if the others were not available;

**^d^**BMI-SDS is a normalized version of BMI expressed as standard deviation score that includes information on age and gender; the results were similar if age and gender were included as covariates;

**^e^**by inverse normal method (function metagen in the package meta of R) with weights proportional to the sample size (fixed effects model).

In sum, our hypothesis-free step-wise design revealed three known (*FTO, MC4R* and *TMEM18*) and two new loci (*SDCCAG8* and *TNKS/MSRA*) with estimated odds ratios that ranged from ∼1.07 to ∼1.44 in children and adolescents and from ∼1.17 to ∼1.45 in adults with the strongest overall signals related to the *FTO* locus. Modelling of the joint and epistatic effects revealed that <1% of the BMI (or BMI-SDS when BMI is expressed as standard deviation score) variance can be attributed to the five variants analyzed in or near *TNKS/MSRA, SDCCAG8, TMEM18, FTO,* and *MC4R*. For children and adolescents this value did not change upon inclusion of gender, age and age^2^ as covariates whereas it changed to 11% for the adult sample (KORA S2-S4). Applying the model including the same covariates derived in one population-based data set of adults (KORA S2-S4) to a second population-based data sets of adults (Heinz-Nixdorf Recall Study) r^2^ dropped from 11% to ∼2%. Proceeding similarly for epistatic effects, we found no evidence for strong epistatic effects using regression tree analyses (Figure S3, Figure S4).

In addition to our hypothesis-free step-wise design, we investigated our paediatric extreme obesity GWAS meta-analysis data focussing on recently reported GWAS-based candidate markers [9], [13], [14]. For the 16 confirmed genetic loci for which quality controlled genotyped or imputed SNPs were available, two loci on chromosome 1 (1p31.1–*NEGR1*, 1q25.2 - *SEC16B*), a locus on 11p14.1 near *BDNF*, and a gene-rich locus on 12q13.13 near *BCDIN3D* all showed directionally consistent effects of the respective SNPs (all p≤.005). Details on all analysed candidate gene SNPs are provided in Table S4 and Table S5. Note that the 16 confirmed genetic loci [9], [13], [14] correspond to 46 SNPs in our GWAS meta-analysis; in case of multiple markers at the same locus all showed evidence for strong LD (r^2^>.9).

## Discussion

We identified two new genomic loci associated with paediatric obesity on chromosomes 1q43–q44 and 8p23.1 by a meta-analysis of two GWAS for early onset extreme obesity with a total 2,258 individuals of European origin. In addition, we confirmed the three known loci *FTO, MC4R* and *TMEM18* using a hypothesis-free step-wise design. Leaving the hypothesis-free approach and focussing on known GWAS-based candidate markers, we were able to substantiate another four loci (*NEGR1*, *SEC16B*, *BDNF* and *BCDIN3D*) of the 16 obesity loci previously detected in GWAS [6], [9], [13], [14]. Thus, we demonstrate that the currently known major common variants related to obesity overlap to a substantial degree between children and adults confirming previous observations for *FTO, MC4R, TMEM18, NEGR1* [2], [6], [14] and extending this observation to *SEC16B*, *BDNF* and *BCDIN3D*; [13], [14]. As our meta-analysis includes data from Meyre et al. [9] an independent well-powered replication of *NPC1*, *MAF* and *PTER* was not possible here.

The new chromosome 1q43–q44 locus was represented by three SNPs in strong pairwise LD (r^2^>.9) which are located in introns 6, 9 and 10 of *SDCCAG8*. There is no obvious indication for an involvement of *SDCCAG8* in body weight regulation. Data on this gene are scarce. It has been shown that SDCCAG8 is located in centrosomes during interphase and mitosis in human and murine cells. N- and C- terminal truncations of the human protein alter this location; a possible role of SDCCAG8 (alternative name: NY-CO-8) in centrosomal organization has been suggested [15]. It is considered to be a naturally occurring autoantigen [16]. *SDCCAG8* is ubiquitously expressed, amongst other tissues in thymus, small intestine, colon mucosa, liver and brain (http://www.genecards.org/cgi-bin/carddisp.pl?gene=SDCCAG8). Hypothalamus, pituitary and adrenals have been shown to have a particularly high transcript abundance. This pattern indicates a role of SDCCAG8 in this pivotal hormonal axis that is well-known for its impact on body weight regulation [16]. Other candidate genes in proximity of the three SNPs include *CEP170* (centrosomal protein 170 kDa gene, ∼95 kb downstream of rs12145833) and *AKT3* (v-akt murine thymoma viral oncogene homolog 3 (protein kinase B, gamma) gene, ∼168 kb upstream of rs12145833) with the latter being the more interesting candidate. The protein encoded by this gene is a member of the AKT family known to regulate cell signalling in response to insulin and growth factors. In particular AS160, an Akt substrate of 160 kDa, and TBC1D1 (TBC1 domain family, member 1) have been suggested to have complementary roles in regulating vesicle trafficking in response to insulin [17] with *TBC1D1* being persuasively linked to body weight regulation [18]–[20]. However, we observed no evidence for strong pairwise LD (r^2^>.9) to any likely functional relevant variant in a region of ±1 Mb around the lead SNP (rs12145833) using Ensembl (version 56; GRCh37, 02/2009; Figure S6).

The new chromosome 8p23.1 locus, for which we observed genome-wide significance in our GWAS meta-analysis (Figure 1, ), was also represented by three SNPs with strong pairwise LD (r^2^>.9). *TNKS* and *MSRA* are the genes located closest to our association finding. *MSRA* encodes a repair enzyme for oxidative damage in proteins by enzymatic reduction of methionine sulfoxide. Oxidation of methionine residues in proteins is considered to be an important consequence of oxidative damage to cells [21]. Oxidation of proteins by reactive oxygen species (ROS) is generally associated with oxidative stress, aging and many neurodegenerative diseases such as Alzheimer's disease [21]. Also, obesity is associated with oxidative stress in the mitochondrion, with the chronic excess of ROS resulting in mitochondrial dysfunction in liver and skeletal muscle contributing to insulin resistance [22]. *MSRA* is mainly expressed in kidney followed by liver, brain, and adipose tissue (http://biogps.gnf.org/#goto=genereport&id=4482). The other candidate gene at the chromosome 8p23.1 locus is *TNKS* which is ubiquitously expressed (http://biogps.gnf.org/#goto=genereport&id=8658). Tankyrase is a Golgi-associated poly-ADP-ribose polymerase, which is involved in the regulation of GLUT4 trafficking in 3T3-L1 adipocytes. Mice lacking Tnks show increased energy expenditure, fatty-acid oxidation, and insulin-stimulated glucose utilization; they are lean even with excessive food intake [23]. In other GWAS, the 8p23.1 genomic region has been related to increased triglyceride levels [24] and to waist circumference in adults [21]. The variants with the strongest reported association signals (rs7819412; rs7826222 which is now labelled rs545854) are about 1.3 and .08 Mb downstream of our best finding (rs473034). For the former, the association to obesity was moderate in our GWAS meta-analysis data (p = 0.02) whereas for the latter no genotype data were available (with pairwise LD between rs545854 and rs473034 of r^2^<.01 (D' = .03) according to Ensembl version 56). Thus, further research is needed to elucidate if our finding for *TNKS*/*MSRA* detected in paediatric extremes of the quantitative trait BMI and the finding for waist circumference in adults [21] point to the same underlying genetic mechanism.

In our study we used two steps to enable hypothesis-free SNP identification and confirmation covering the extremes and the population distribution of BMI in paediatric as well as adult samples. Both dimensions of our design are related to statistical power considerations and the genetic architecture of the phenotype studied. A case-control design with highly selected individuals outperforms a design using unselected population-based individuals if the same number of individuals are genotyped and if the same alternative hypothesis holds true (see Text S1). This contrast will be aggravated the more extreme the selection and possibly also the younger the subjects [25]. In addition the selection of extremes may lead to the detection of genetic variations that are rare in the population, that accumulated in families and that might result in stronger effect sizes. Nevertheless, the power of our GWAS meta-analysis sample is still limited for small effects (see Text S1) and growing consortia like GIANT [14] will be best suited to detect them. Not surprisingly, we confirmed the strongest effects (odds ratio for the obesity risk effect alleles of ∼1.4) reported for children and adolescents near *FTO, MC4R* and *TMEM18* [12] but also found support for variants near *NEGR1*, *SEC16B, BDNF* and *BCDIN3D.* Thus, one may speculate, that the genetic architecture in the paediatric extremely obese is in part similar to the BMI findings based mainly on adults from large population-based assessments (e.g. [13], [14]). On the other hand, some of the related effect sizes of these variants seem to vary longitudinally as shown here for *MC4R* and previously stressed by others [6], [26] while other genetic loci might only be relevant for (paediatric) extreme obesity such as *TNKS/MSRA.*

In conclusion, two new loci related to body weight regulation were identified using highly selected paediatric samples from the extremes of the quantitative phenotype BMI. By showing that one locus is relevant across all age groups whereas the impact of a second is limited to childhood and adolescence, our data support previous studies showing the importance of age-related aspects upon interpretation of GWAS signals.

## Materials and Methods

### Study samples, genotyping, and quality control

Our study design consisted of two steps (Figure 1). As first part of the DISCOVERY step we performed a meta-analysis of two genome-wide association studies (GWAS) including 1,370 individuals of French and 888 of German ancestry, defined by self-reported ethnicity. Ascertainment in both GWAS was very similar with a focus on extremely obese children and adolescents and normal weight or lean controls (Table S1). Body-mass-index (BMI in kg/m^2^) was calculated and the extremes were defined using percentile criteria of large population-based samples of the general population [27], [28]. We applied the cut-offs ≥97th percentile and ≥90th percentile to define ‘obesity’ and ‘overweight’ in children and adolescents; most of the cases with extreme obesity had a BMI ≥99th percentile (Table S1; [29]). Whole-genome genotyping was carried out using the Illumina Human CNV370-Duo array (French GWAS) and the Affymetrix Genome-Wide Human SNP Array 6.0 (German GWAS). Genotype data quality measures, e.g. genotype calling rates, were similar in both GWAS (Table S2). To combine both datasets, the GWAS genotypes were imputed using publicly available HapMap CEU (release 22; http://www.hapmap.org). From this GWAS meta-analysis, we selected 44 SNPs covering 21 loci (Table S3; Figure S5) which we (*de novo*) genotyped in 1,181 overweight and obese children and adolescents and 1,960 normal weight or lean children and adolescents and young adults (controls) of European ancestry and up to 715 nuclear families with obese offspring of European ancestry were examined. The SNP selection was based on (i) an unadjusted two-sided p-values ≤10^−5^ and (ii) more than a single SNP within a locus (lead SNP ±500 kb) showing evidence for association (with a p-value rank <1,500 roughly corresponding to p≤5×10^−4^; for details see Text S1). Sub-whole genome SNP genotyping was performed using by the MALDI-TOF mass spectrometry-based iPLEX Gold assay. In the GENERALIZATION step, 10 SNPs, for which DISCOVERY step had revealed consistent observations (Table 1; Table 2), were further investigated for generalizability to adults and to unselected population-based samples. Thus, 711 overweight and obese children and adolescents (Datteln Paediatric Obesity sample), 3,525 children and adolescents from the general population (GINI, LISA, Berlin School Girls), 988 obese adults (Marburg Adult Obesity sample) and 25,958 adults from the general population (EPIC-Potsdam Study, KORA S2-S4, SHIP, Heinz-Nixdorf Recall Study) each of European ancestry were genotyped. SNP genotyping was performed by the MALDI-TOF mass spectrometry-based iPLEX Gold assay at the Helmholtz Zentrum, München and at the Department of Genomics, Life & Brain Center, Bonn or by KBioscience, Hoddeston, UK. All were assessed for genotype calling rates and deviations from Hardy–Weinberg equilibrium (for details see Text S1).

The RefSeq accession numbers for the reported genes are: *FTO*: NM_001080432; *MC4R*: NM_005912; *TNKS*: NM_003747; *SDCCAG8*: NM_006642.2; *TMEM18*: NM_152834; *CEP170*: NM_014812; *AKT3*: NM_181690.

### Statistical analysis

After similar quality control analyses of both GWAS, the imputed GWAS were jointly analysed using the inverse normal method to combine p-values of allele-based chi-square tests. Details on the imputation and on the marker selection for the follow-up are described in Text S1. In the paediatric extreme obesity GWAS meta-analysis data set we also explored genetic variants for obesity recently derived from other GWAS [9], [13], [14] and variants for ‘classical’ obesity candidate genes [3], [30] by testing the best SNP reported in Scuteri et al. [4].

In both the DISCOVERY and the GENERALIZATION part of the study either log-additive or additive genetic models were applied. Case-control samples were analysed using logistic regression (both with and without gender and age as covariates). The nuclear families were analysed using UNPHASED (Version 3.0.13; [31]) which addresses the correlation among sibs and provides estimators; nuclear family data and case-control data sets were combined using a method described in [32]. In the GENERALIZATION step, BMI in adults of population-based samples was analysed using linear regression with gender and age as covariates. Similarly, we used linear regression analyses for the population-based samples of children and adolescents. However, as phenotype we used a normalized version of the BMI applying Cole's least mean square method [33] to express BMI as a standard deviation score (BMI-SDS) which is comparable to the BMI z-score as e.g. used by the Center for Disease Control and Prevention (http://www.cdc.gov/). As BMI-SDS already includes information on gender and age additional sensitivity analyses were performed where these covariates were omitted. Note that the case-control analyses in GENERALIZATION step are not completely independent from the population-based analyses. In particular, controls in GENERALIZATION were individuals from the population-based samples which either had a BMI<25 for adults or a BMI percentile below the median. Due to the similarity to the original design it was nevertheless decided to report both analyses.

As secondary sensitivity analyses, we performed gender stratified analyses in all GENERALIZATION samples for the markers which we followed-up. We explored the recessive and dominant genetic model, investigated the impact of the control group cut-off for the case-control analyses (results not shown as they did not alter the conclusions drawn here) and explored joint and epistatic effects (multiple linear regression and regression trees using lm, rlm, and party of R.2.9.1) of all five loci (see Figure S3, Figure S4). To address, to some extent, problems of the ‘bias-variance trade-off’ and the ‘winners curse’ [34], the largest GENERALIZATION population-based sample KORA (n = 12,002) was chosen for this modelling. The model was tested in the Heinz-Nixdorf Recall Study sample (n = 4,646). These two samples were chosen due to their largest similarities in the recruitment and due to the availability of directly genotyped SNPs. In addition, we also explored the sample of population-based children and adolescents (GINI, LISA, Berlin School Girls; n = 3,525) separately.

Unless otherwise stated, all reported p-values are nominal, two-sided and not adjusted for multiple testing. To address multiple testing in the paediatric extreme obesity GWAS meta-analysis we applied a Bonferroni-corrected α_BF_≈3.1×10^−8^ to the quality controlled SNPs on autosomes. Confidence intervals were calculated with coverage of 95% (abbreviated 95%CI). More details on quality control and power considerations are provided in Text S1.

### Ethics statement

The study, including the protocols for subject recruitment and assessment, the informed consent for participants, were reviewed and approved by all local IRB boards.

## Supporting Information

Figure S1 DISCOVERY: Quantile-quantile plot of SNPs of the GWAS meta-analysis focussing on extremely obese children and adolescents joint analysis (grey unadjusted; black adjusted results - for details on the adjustment see Text S1). The deviation from the 45-degree-line is due to the presence of multiple truly associated markers, the ascertainment of the study samples and in part due to the strategy of the combination for C/G or A/T SNPs.
(4.19 MB TIF)

Figure S2 Regional plots of two new loci associated with obesity. The SNPs are plotted on the x-axis according to their position on each chromosome (HapMap, release 22) against the meta-analysis association signal on the y-axis (shown as -log_10_ of the two-sided p-value). The plots were generated using SNAP ([24] of Text S1).
(8.26 MB TIF)

Figure S3 GENERALIZATION: Regression trees to explore epistatic effects of validated markers in two independent population-based samples of adults (left: KORA; right: Heinz-Nixdorf Recall Study; see main text and Text S1 for details). Only the five loci of main paper were modelled. Splits in the branches of the tree indicate different risk classes starting with the strongest predictor. Here the samples are first split by *FTO* genotype and then by *TMEM18* genotype. Here we observe some weak evidence for a marker by marker interaction as the sub-branching in the *FTO* genotype branches is not the same for both branches.
(9.26 MB TIF)

Figure S4 GENERALIZATION: Regression trees to explore epistatic effects of validated markers in one population-based sample of children and adolescents (GINI, LISA, Berlin School Girls; left: modelling of the five loci only; right: modelling of the five loci plus sex, age and age ^2^; see main text and Text S1 for details). Splits in the branches of the tree indicate different risk classes starting with the strongest predictor. Here the samples are first split by *FTO* genotype and then by *MC4R* genotype. However, as shown on the right panel, if age (regression tree based cut-off at 13.19 years) is included only the *FTO* genotype remains as predictor.
(9.42 MB TIF)

Figure S5 DISCOVERY: 21 regions of interest from the meta-analysis of two genome-wide association studies for early onset extreme obesity. Displayed are the number of SNPs per region for all 213 SNPs with an unadjusted two-sided p-values ≤10^−5^ (see Text S1 for details).
(4.19 MB TIF)

Figure S6 Regional plots of the new chromosome 1q43–q44 locus located in *SDCCAG8*. All variants of Ensembl (version 56; GRCh37, 02/2009) in a region of ± 1Mb around the lead SNP (rs12145833) are displayed. The x-axis displays the chromosomal position of the variant whereas the y-axis indicates LD (r^2^) of that variant with rs12145833; the different colours code for different variant classes (see legend). The plots were generated using CandiSNPer ([25] of Text S1).
(5.52 MB TIF)

Table S1 DISCOVERY: Description of samples that were jointly analysed in our genome-wide association analysis.
(0.05 MB DOC)

Table S2 DISCOVERY: Genotype data for both GWAS in extreme early onset obesity.
(0.04 MB DOC)

Table S3 DISCOVERY: Evidence from obese children and adolescents (n = 1,181) versus controls (n = 1,960) and 715 nuclear families with obese offspring. All these samples were not part of the meta-analysis of two GWAS for early onset extreme obesity.
(0.20 MB DOC)

Table S4 DISCOVERY: GWAS-based SNPs of previously reported candidate markers for BMI and/or obesity sorted by chromosome and physical position. The first two columns indicate the name of a previously identified marker and the implied, described candidate genes (in bold those which were confirmed and which are reported in the introduction of the main text). The columns 6–11 summarize the data of three recently published large-scale GWAS (Willer et al., 2009 (publication “WI” and “WI.b” for the Appendix of “WI.b”), Thorleifsson et al., 2009 (publication “TH”), and Meyre et al., 2009 (publication “ME”)). Note that parts of the data sets in Meyre et al. (2009) overlap with our meta-analyses data set. The table displays the phenotype, obesity risk effect allele, the frequency of the effect allele, the estimated additive effect and the related nominal p-value are derived from publicly available resources. The effect is displayed using the measurement regarded most appropriate for the design of the GWAS. The remaining columns correspond to the respective results observed GWAS meta-analysis.
(0.54 MB DOC)

Table S5 DISCOVERY: SNPs of previously identified ‘classical’ obesity candidate genes. The first column indicates the name of a previously identified candidate gene. The second column indicates SNPs which showed strongest association in Scuteri et al. (2007) for the phenotype, effect allele, frequency, the estimated additive effect and the related nominal p-value in columns 6–9. The remaining columns correspond to the respective results observed in our GWAS meta-analysis (only markers with two-sided adjusted p-values <.1 and the ‘directionally consistent’ obesity risk effect allele are displayed).
(0.07 MB DOC)

Text S1 DISCOVERY and GENERALIZATION.
(0.27 MB DOC)
