# Supplementary figures and images for: Two New Loci for Body-Weight Regulation Identified in a Joint Analysis of Genome-Wide Association Studies for Early-Onset Extreme Obesity in French and German Study Groups
Source: PLoS Genet. 2010 Apr 22;6(4):e1000916. doi: 10.1371/journal.pgen.1000916 (PMC2858696; doi:10.1371/journal.pgen.1000916)

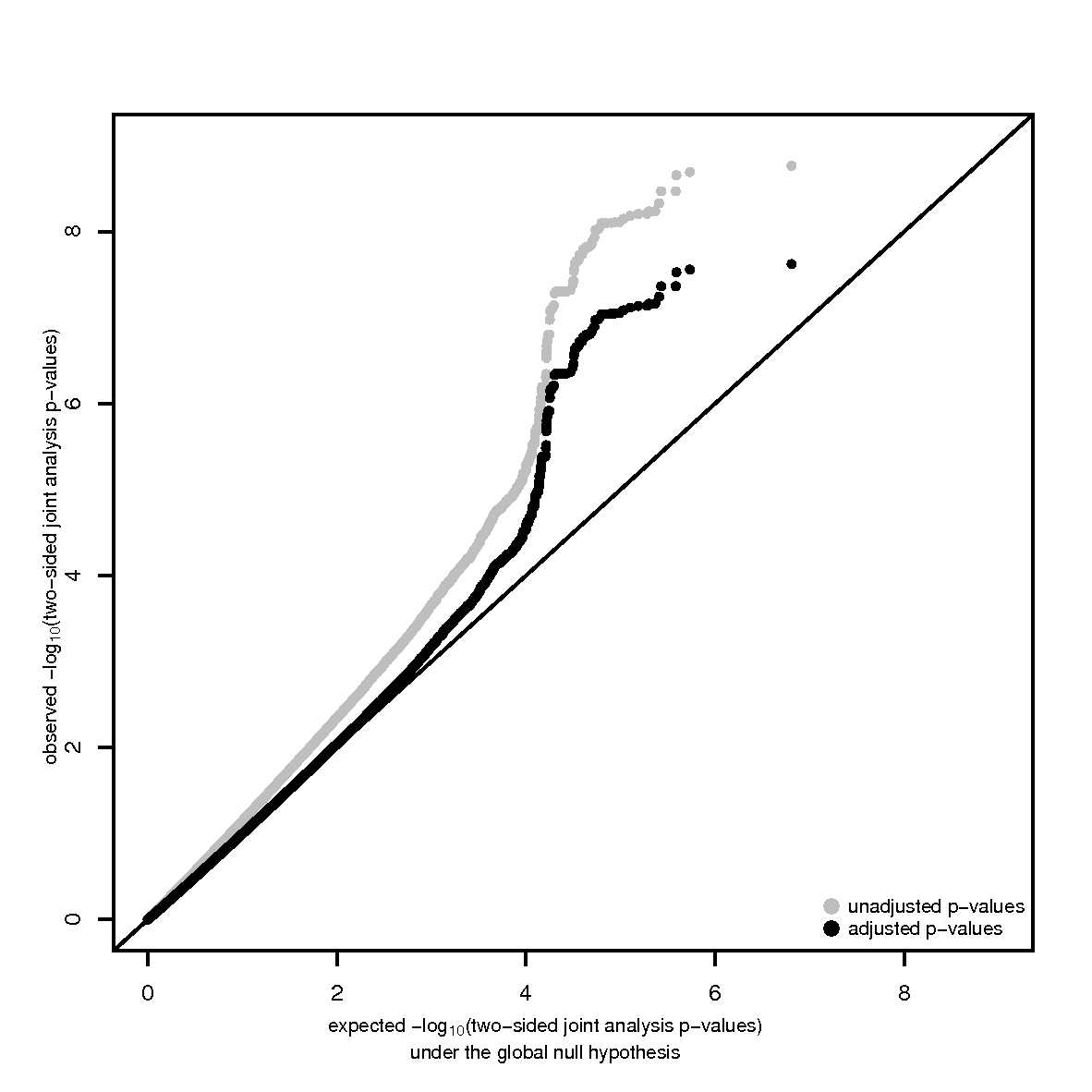

Supplement: Figure S1 — DISCOVERY: Quantile-quantile plot of SNPs of the GWAS meta-analysis focussing on extremely obese children and adolescents joint analysis (grey unadjusted; black adjusted results - for details on the adjustment see Text S1). The deviation from the 45-degree-line is due to the presence of multiple truly associated markers, the ascertainment of the study samples and in part due to the strategy of the combination for C/G or A/T SNPs. (4.19 MB TIF) [file pgen.1000916.s001.tif]

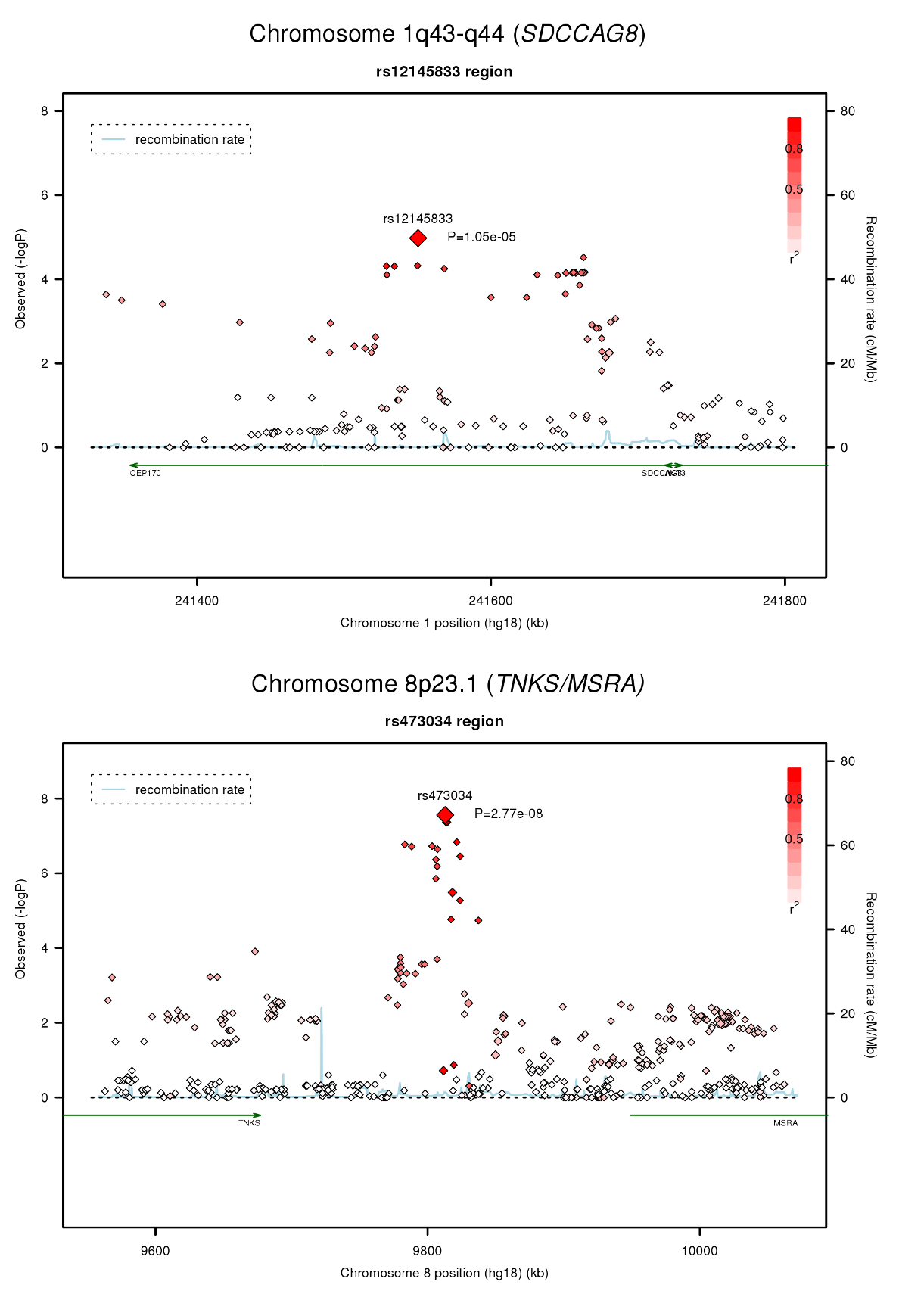

Supplement: Figure S2 — Regional plots of two new loci associated with obesity. The SNPs are plotted on the x-axis according to their position on each chromosome (HapMap, release 22) against the meta-analysis association signal on the y-axis (shown as -log10 of the two-sided p-value). The plots were generated using SNAP ([24] of Text S1). (8.26 MB TIF) [file pgen.1000916.s002.tif]

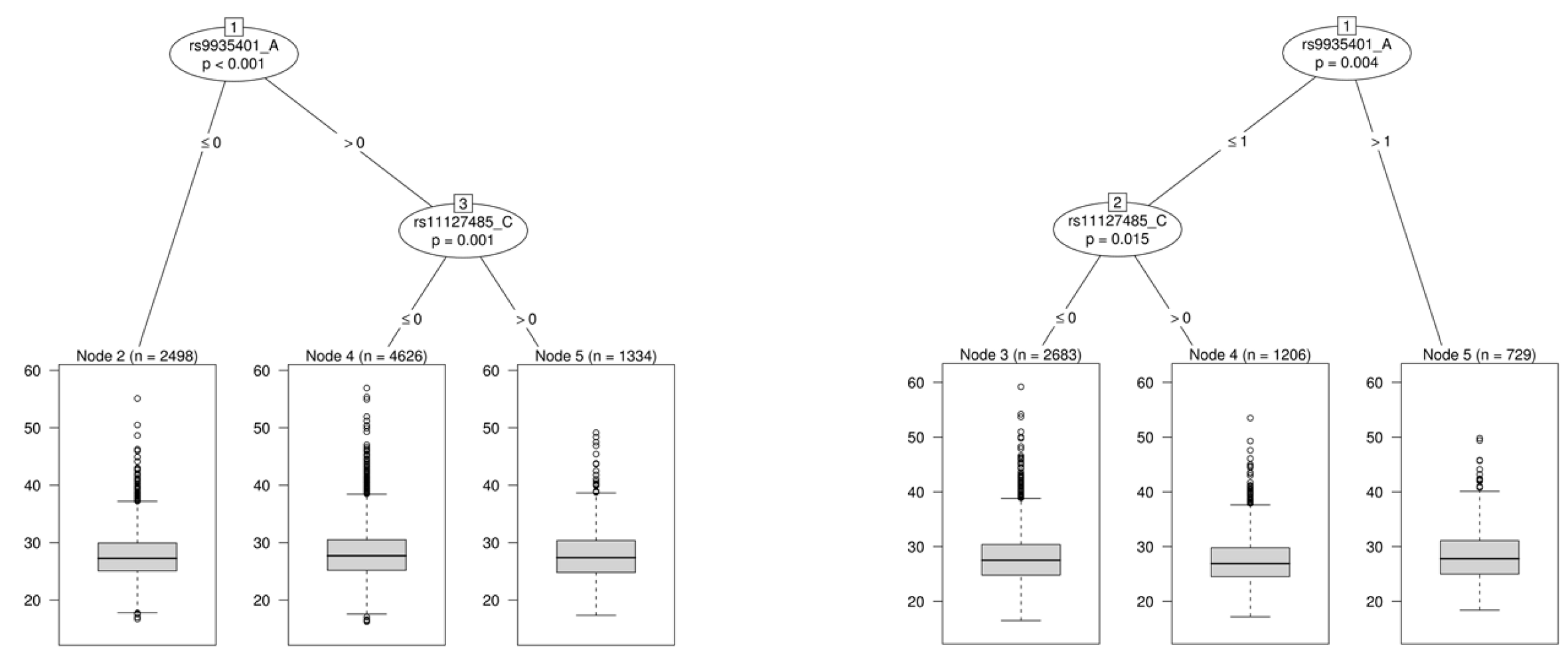

Supplement: Figure S3 — GENERALIZATION: Regression trees to explore epistatic effects of validated markers in two independent population-based samples of adults (left: KORA; right: Heinz-Nixdorf Recall Study; see main text and Text S1 for details). Only the five loci of main paper were modelled. Splits in the branches of the tree indicate different risk classes starting with the strongest predictor. Here the samples are first split by FTO genotype and then by TMEM18 genotype. Here we observe some weak evidence for a marker by marker interaction as the sub-branching in the FTO genotype branches is not the same for both branches. (9.26 MB TIF) [file pgen.1000916.s003.tif]

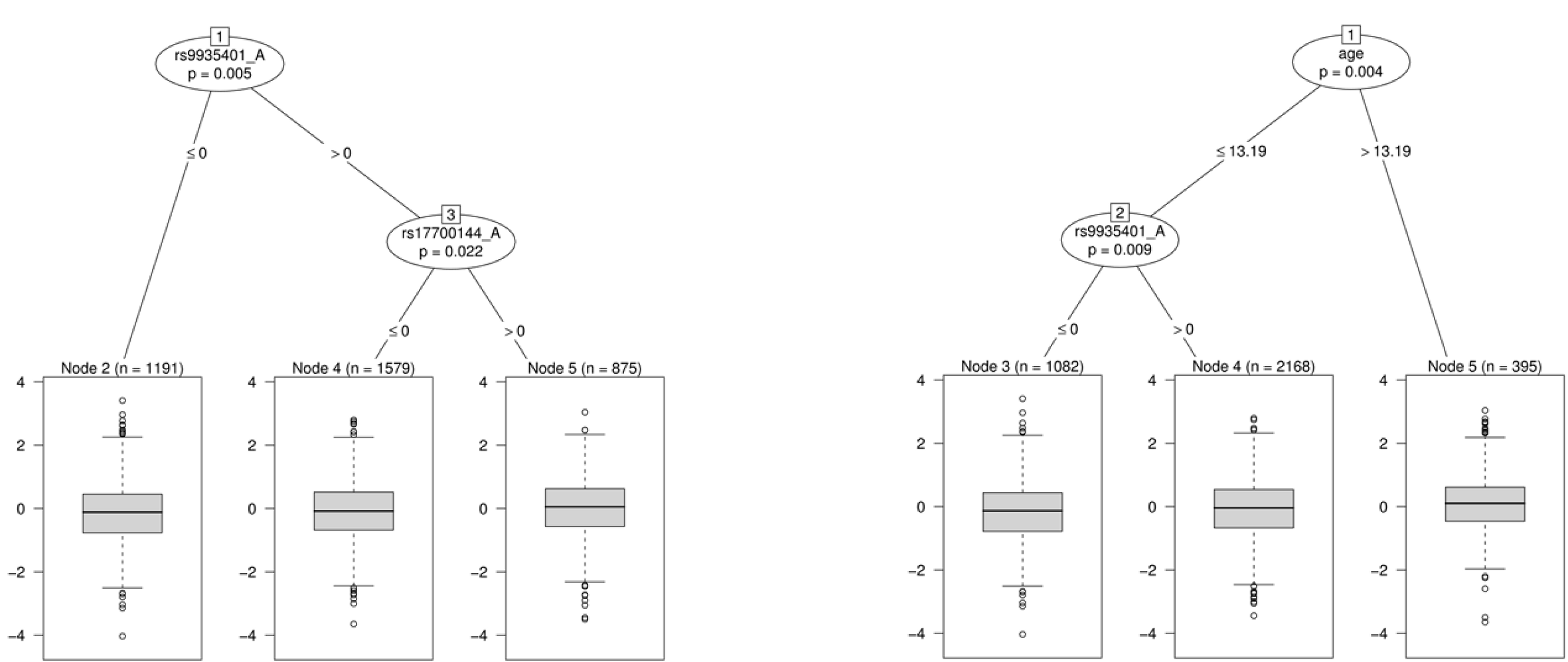

Supplement: Figure S4 — GENERALIZATION: Regression trees to explore epistatic effects of validated markers in one population-based sample of children and adolescents (GINI, LISA, Berlin School Girls; left: modelling of the five loci only; right: modelling of the five loci plus sex, age and age 2; see main text and Text S1 for details). Splits in the branches of the tree indicate different risk classes starting with the strongest predictor. Here the samples are first split by FTO genotype and then by MC4R genotype. However, as shown on the right panel, if age (regression tree based cut-off at 13.19 years) is included only the FTO genotype remains as predictor. (9.42 MB TIF) [file pgen.1000916.s004.tif]

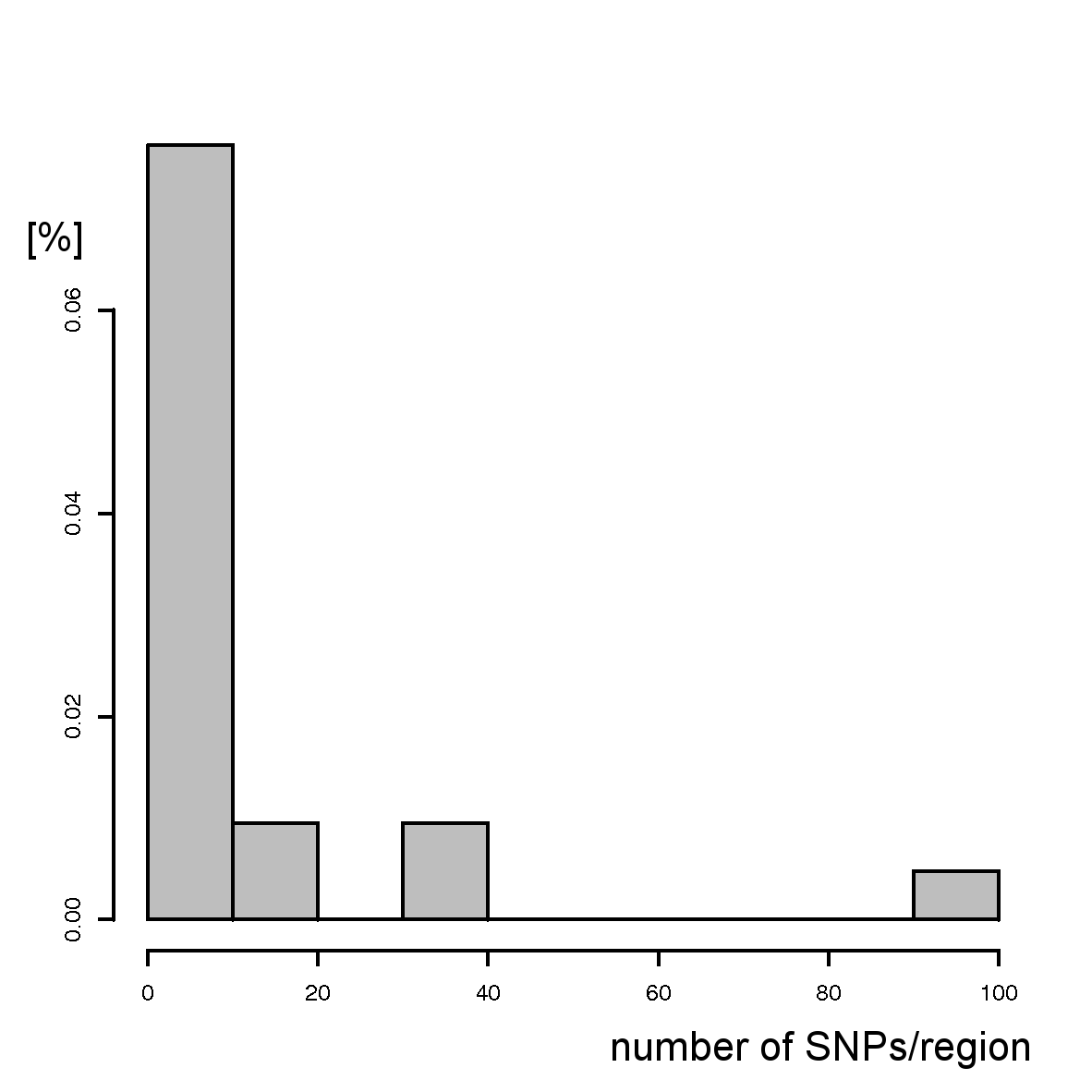

Supplement: Figure S5 — DISCOVERY: 21 regions of interest from the meta-analysis of two genome-wide association studies for early onset extreme obesity. Displayed are the number of SNPs per region for all 213 SNPs with an unadjusted two-sided p-values ≤10−5 (see Text S1 for details). (4.19 MB TIF) [file pgen.1000916.s005.tif]

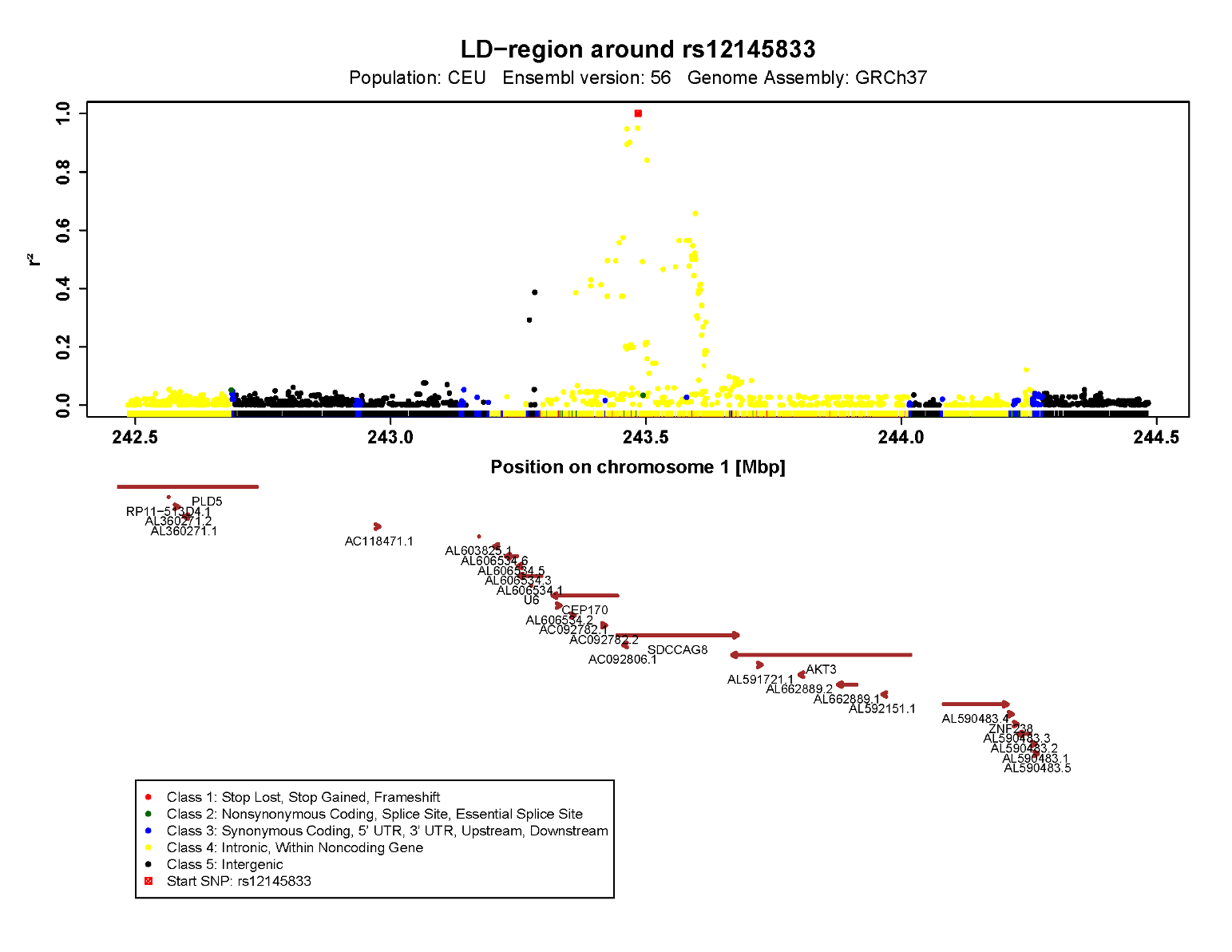

Supplement: Figure S6 — Regional plots of the new chromosome 1q43–q44 locus located in SDCCAG8. All variants of Ensembl (version 56; GRCh37, 02/2009) in a region of ± 1Mb around the lead SNP (rs12145833) are displayed. The x-axis displays the chromosomal position of the variant whereas the y-axis indicates LD (r2) of that variant with rs12145833; the different colours code for different variant classes (see legend). The plots were generated using CandiSNPer ([25] of Text S1). (5.52 MB TIF) [file pgen.1000916.s006.tif]
