# Supplementary material for: Two New Loci for Body-Weight Regulation Identified in a Joint Analysis of Genome-Wide Association Studies for Early-Onset Extreme Obesity in French and German Study Groups
Source: PLoS Genet. 2010 Apr 22;6(4):e1000916. doi: 10.1371/journal.pgen.1000916 (PMC2858696; doi:10.1371/journal.pgen.1000916)
Supplement: Table S1 — DISCOVERY: Description of samples that were jointly analysed in our genome-wide association analysis. (0.05 MB DOC) [file pgen.1000916.s007.doc]

**Table S1.** DISCOVERY: Description of samples that were jointly analysed in our genome-wide association analysis.

|  |  |  |  |  |  |  |
| --- | --- | --- | --- | --- | --- | --- |
| sample | | status | n total  (female) | mean age [years] (SD) | mean BMI [m/kg^2^] (SD) | mean BMI-SDS^a^ (SD) |
|  |  |  |  |  |  |  |
| French GWAS |  |  |  |  |  |  |
|  | obese children and adolescents | cases | 685 (375) | 10.89 (3.27) | 29.47 (6.46) | 4.28 (1.24)^b^ |
|  | healthy normal weight individuals (children and adolescents) | controls | 685 (353) | 11.93 (2.27) | 17.56 (2.33) | -0.14 (0.97) |
| German GWAS |  |  |  |  |  |  |
|  | obese children and adolescents | cases | 453 (261) | 14.37 (3.75) | 33.15 (6.68) | 4.55 (2.16)^c^ |
|  | healthy lean individuals (young adults) | controls | 435 (264) | 26.08 (5.75) | 18.31 (1.11) | -1.38 (0.36) |
|  |  |  |  |  |  |  |

^a^ BMI-SDS is a normalized version of BMI expressed as standard deviation score that includes information on age and gender;

^b^ all had a BMI ≥ 97th percentile according to a French reference population (Rolland-Cachera, M.F et al., 1991)

^c^ 410 (91%) had a BMI ≥ 97th percentile according to [www.mybmi.de](http://www.mybmi.de/) (Kromeyer-Hauschild et al. (2001); according to an older reference set (National Nutrition Survey I) all individuals had a BMI ≥ 97th percentile
