# Supplementary material for: Two New Loci for Body-Weight Regulation Identified in a Joint Analysis of Genome-Wide Association Studies for Early-Onset Extreme Obesity in French and German Study Groups
Source: PLoS Genet. 2010 Apr 22;6(4):e1000916. doi: 10.1371/journal.pgen.1000916 (PMC2858696; doi:10.1371/journal.pgen.1000916)
Supplement: Table S2 — DISCOVERY: Genotype data for both GWAS in extreme early onset obesity. (0.04 MB DOC) [file pgen.1000916.s008.doc]

**Table S2.** DISCOVERY: Genotype data for both GWAS in extreme early onset obesity.

|  | French GWAS Illumina Human CNV370-Duo array | German GWAS  Affymetrix ® Genome-Wide Human SNP Array 6.0 |
| --- | --- | --- |
| **# markers** | 318,237 | 909,622 |
| **#cases/controls** | 685/685 | 453/435 |
|  |  |  |
| **#markers removed for**^a^**:** |  |  |
| call rate^b^ | 1,622 | 34,373 |
| MAF^c^ | 281 | 143,876 |
| HWE^d^ | 1,662 | 4,726 |
| unknown position | -- | 3,001 |
|  |  |  |
| **#markers that pass QC** | 308,846 | 730,577 |
|  |  |  |
| **total genotping rate** | 99.54% | 99.16 % |

^a^note that some markers were removed by more than one filter

^b^Call rate per individual and call rate per marker ≤95%

^c^Minor allele frequency >=1%

^d^exact two-sided HWE test with a threshold of p ≤ .001 in the controls
