# Supplementary material for: Two New Loci for Body-Weight Regulation Identified in a Joint Analysis of Genome-Wide Association Studies for Early-Onset Extreme Obesity in French and German Study Groups
Source: PLoS Genet. 2010 Apr 22;6(4):e1000916. doi: 10.1371/journal.pgen.1000916 (PMC2858696; doi:10.1371/journal.pgen.1000916)
Supplement: Table S3 — DISCOVERY: Evidence from obese children and adolescents (n = 1,181) versus controls (n = 1,960) and 715 nuclear families with obese offspring. All these samples were not part of the meta-analysis of two GWAS for early onset extreme obesity. (0.20 MB DOC) [file pgen.1000916.s009.doc]

**Table S3.** DISCOVERY: Evidence from obese children and adolescents (n=1,181) vs. controls (n=1,960) and 715 nuclear families with obese offspring. All these samples were not part of the meta-analysis of two GWAS for early onset extreme obesity.

| Chromo-somal region^a^  -  suggested gene | SNP | Chromosome (Position^b^) | obesity risk effect allele^c^ | heterogeneity  measure  I^2^  [%] | obese children and adolescents vs. controls | | | | | |  | nuclear families with obese offspring | | |
| --- | --- | --- | --- | --- | --- | --- | --- | --- | --- | --- | --- | --- | --- | --- |
|  |  |  |  |  | French  (537 cases vs. 566 controls) | | German (389 cases vs. 1,135 controls) | | Finnish (255 cases vs. 259 controls) | |  |  |  |  |
|  |  |  |  |  | effect (oddsratio) | p-value | effect (oddsratio) | p-value | effect (oddsratio) | p-value |  | effect^d^ | (95% CI)^d^ | p-value |
|  | rs10489964 | 1 (180,889,972) | C | 36.8 | 0.97 | 0.77 | 0.89 | 0.26 | 1.24 | 0.09 |  | 0.91 | (0.74;1.13) | 0.39 |
| 1q43-q44  -  *SDCCAG8* | rs10926984 | 1  (241,528,776) | T | 0.0 | 1.30 | 0.08 | 1.22 | 0.17 | 0.92 | 0.62 |  | 1.19 | (0.98;1.45) | 0.07 |
|  | rs12145833 | 1  (241,550,377) | T | 23.2 | 1.41 | 0.02 | 1.16 | 0.27 | 0.92 | 0.62 |  | 1.23 | (1.01;1.52) | 0.04 |
|  | rs2783963 | 1  (241,568,206) | C | 9.9 | 1.15 | 0.33 | 1.16 | 0.31 | 0.88 | 0.44 |  | 1.25 | (1.02;1.52) | 0.03 |
| 2p25.3  -  *TMEM18* | rs11127485 | 2  (622,028) | T | 62.7^e^ | 1.67 | 2.9x10^-4^ | 0.98 | 0.87 | 1.12 | 0.45 |  | 1.27 | (1.03;1.54) | 0.02 |
|  | rs2011946 | 2  (136,534,086) | T | -- | -- | -- | -- | -- | -- | -- |  | 1.04 | (0.84;1.28) | 0.67 |
|  | rs17304995 | 3  (53,045,795) | G | 0.0 | 1.04 | 0.71 | 1.04 | 0.74 | 1.09 | 0.50 |  | 1.01 | (0.82;1.23) | 0.96 |
|  | rs2968959 | 4  (131,046,586) | A | -- | -- | -- | -- | -- | -- | -- |  | 0.78 | (0.62;0.98) | 0.03 |
|  | rs2391510 | 4  (131,052,029) | G | 25.3 | 1.23 | 0.05 | 1.04 | 0.70 | 1.23 | 0.11 |  | 0.95 | (0.77;1.16) | 0.61 |
|  | rs1995720 | 4  (131,107,101) | T | 0.0 | 1.14 | 0.21 | 1.03 | 0.74 | 1.25 | 0.07 |  | 1.03 | (0.85;1.25) | 0.77 |
|  | rs2968941 | 4  (131,118,918) | C | -- | -- | -- | -- | -- | -- | -- |  | 1.07 | (0.84;1.36) | 0.58 |
|  | rs2968937 | 4  (131,125,038) | C | 29.3 | 1.27 | 0.01 | 1.03 | 0.79 | 1.32 | 0.01 |  | 1.10 | (0.91;1.34) | 0.32 |
|  | rs2391576 | 4  (131,146,911) | G | 24.9 | 1.30 | 0.01 | 1.10 | 0.38 | 1.24 | 0.09 |  | 0.99 | (0.81;1.22) | 0.96 |
|  | rs1567191 | 4  (131,169,458) | G | 57.6 | 1.31 | 0.01 | 1.10 | 0.38 | 1.22 | 0.11 |  | 0.89 | (0.72;1.11) | 0.32 |
|  | rs1830876 | 6 (51,173,716) | C | -- | -- | -- | -- | -- | -- | -- |  | 0.93 | (0.77;1.13) | 0.48 |
|  | rs4142510 | 6  (119,145,811) | T | 56.0 | 0.91 | 0.35 | 0.93 | 0.42 | 1.33 | 0.02 |  | 0.96 | (0.81;1.16) | 0.71 |
|  | rs9375168 | 6  (123,073,201) | A | -- | -- | -- | -- | -- | -- | -- |  | 0.92 | (0.75;1.12) | 0.42 |
|  | rs1859090 | 7  (125,448,739) | G | -- | -- | -- | -- | -- | -- | -- |  | 0.98 | (0.80;1.20) | 0.87 |
|  | rs10487432 | 7  (125,458,829) | C | 85.3^e^ | 1.18 | 0.13 | 0.72 | 1.3x10^-3^ | 1.35 | 0.01 |  | 0.86 | (0.69;1.08) | 0.19 |
|  | rs3898382 | 7  (125,480,338) | C | -- | -- | -- | -- | -- | -- | -- |  | 1.02 | (0.81;1.30) | 0.86 |
|  | rs12706717 | 7  (125,527,081) | A | 85.8^e^ | 1.28 | 0.02 | 0.74 | 2.6x10^-3^ | 1.33 | 0.01 |  | 0.93 | (0.73;1.16) | 0.51 |
| 8p23.1  -  *TNKS / MSRA* | rs17150703 | 8  (9,783,208) | A | 65.3^e^ | 1.05 | 0.78 | 1.65 | 1.2x10^-3^ | 0.91 | 0.50 |  | 1.22 | (0.98;1.53) | 0.07 |
|  | rs13278851 | 8  (9,788,282) | A | -- | -- | -- | -- | -- | -- | -- |  | 1.29 | (1.04;1.61) | 0.02 |
|  | rs516175 | 8  (9,806,983) | T | 51.6 | 1.18 | 0.30 | 1.57 | 2.2x10^-3^ | 0.97 | 0.84 |  | 1.09 | (0.89;1.34) | 0.38 |
|  | rs636817 | 8  (9,807,279) | G | -- | -- | -- | -- | -- | -- | -- |  | 1.17 | (0.81;1.68) | 0.41 |
|  | rs473034 | 8  (9,812,926) | A | 47.8 | 1.02 | 0.88 | 1.39 | 0.02 | 0.95 | 0.72 |  | 1.40 | (1.04;1.88) | 0.03 |
|  | rs587162 | 8  (9,813,504) | C | -- | -- | -- | -- | -- | -- | -- |  | 1.40 | (1.04;1.87) | 0.02 |
|  | rs1660627 | 10  (34,510,310) | T | 0.0 | 1.10 | 0.42 | 1.03 | 0.75 | 0.98 | 0.92 |  | 1.08 | (0.88;1.33) | 0.46 |
|  | rs1660628 | 10  (34,510,760) | C | 0.0 | 1.10 | 0.39 | 1.03 | 0.80 | 0.97 | 0.86 |  | 1.12 | (0.89;1.41) | 0.33 |
|  | rs6485700 | 11  (46,832,509) | G | -- | -- | -- | -- | -- | -- | -- |  | 1.03 | (0.73;1.47) | 0.86 |
|  | rs7112060 | 11  (46,848,477) | T | -- | -- | -- | -- | -- | -- | -- |  | 1.03 | (0.72;1.48) | 0.86 |
|  | rs10437892 | 12  (101,629,051) | T | 0.0 | 1.00 | 0.98 | 1.04 | 0.70 | 1.14 | 0.27 |  | 0.98 | (0.80;1.19) | 0.84 |
|  | rs604192 | 13  (97,772,864) | A | -- | -- | -- | -- | -- | -- | -- |  | 1.05 | (0.85;1.28) | 0.68 |
|  | rs2288604 | 15  (31,836,907) | T | 29.9 | 1.16 | 0.23 | 0.88 | 0.28 | 0.83 | 0.13 |  | 0.97 | (0.76;1.23) | 0.81 |
| 16q12.2  -  *FTO* | rs1558902 | 16  (52,361,075) | A | 45.5 | 1.34 | 2.8x10^-3^ | 1.65 | 8.2x10^-3^ | 1.37 | 4.4x10^-3^ |  | 1.26 | (1.10;1.45) | 8.6x10^-4^ |
|  | rs9935401 | 16  (52,374,339) | A | -- | -- | -- | -- | -- | -- | -- |  | 1.20 | (1.05;1.38) | 0.01 |
|  | rs11644513 | 16  (81,426,353) | C | 3.6 | 1.20 | 0.09 | 0.99 | 0.95 | 1.16 | 0.20 |  | 0.97 | (0.79;1.19) | 0.76 |
|  | rs918657 | 16  (81,428,851) | G | -- | -- | -- | -- | -- | -- | -- |  | 1.12 | (0.91;1.39) | 0.28 |
|  | rs9319575 | 16  (81,432,231) | A | 37.3 | 1.30 | 0.02 | 1.01 | 0.93 | 1.12 | 0.32 |  | 0.94 | (0.76;1.16) | 0.56 |
|  | rs10502296 | 18  (1,937,102) | G | 69.3^e^ | 1.52 | 4.1x10^-3^ | 0.98 | 0.87 | 0.82 | 0.18 |  | 1.14 | (0.92;1.39) | 0.22 |
|  | rs1941706 | 18 (29,479,028) | C | -- | -- | -- | -- | -- | -- | -- |  | 1.08 | (0.89;1.30) | 0.45 |
| 18q21.32  -  *MC4R* | rs663129 | 18  (55,989,381) | T | -- | -- | -- | -- | -- | -- | -- |  | 0.99 | (0.79;1.25) | 0.95 |
|  | rs571312 | 18  (55,990,749) | T | -- | -- | -- | -- | -- | -- | -- |  | 1.24 | (1.00;1.52) | 0.04 |
|  | rs10871777 | 18  (56,002,743) | G | 56.5 | 0.98 | 0.88 | 1.42 | 8.7x10^-4^ | 1.39 | 0.02 |  | 1.19 | (0.96;1.48) | 0.10 |

^a^ only regions and suggested genes of the GENERALIZATION are provided; ^b^ position and stranding according to dbSNP BUILD 129 (<http://www.ncbi.nlm.nih.gov/SNP/>); Map to Genome Build 36.3; ^c^ obesity risk effect alleles as derived from the GWAS meta-analysis; ^d^ derived from UNPHASED 3.0 - a 95% confidence interval is added as an indicator for the number of informative families; ^e^ p-values ≤.05 as derived from the package meta of R
