# Supplementary material for: Two New Loci for Body-Weight Regulation Identified in a Joint Analysis of Genome-Wide Association Studies for Early-Onset Extreme Obesity in French and German Study Groups
Source: PLoS Genet. 2010 Apr 22;6(4):e1000916. doi: 10.1371/journal.pgen.1000916 (PMC2858696; doi:10.1371/journal.pgen.1000916)
Supplement: Table S4 — DISCOVERY: GWAS-based SNPs of previously reported candidate markers for BMI and/or obesity sorted by chromosome and physical position. The first two columns indicate the name of a previously identified marker and the implied, described candidate genes (in bold those which were confirmed and which are reported in the introduction of the main text). The columns 6–11 summarize the data of three recently published large-scale GWAS (Willer et al., 2009 (publication “WI” and “WI.b” for the Appendix of “WI.b”), Thorleifsson et al., 2009 (publication “TH”), and Meyre et al., 2009 (publication “ME”)). Note that parts of the data sets in Meyre et al. (2009) overlap with our meta-analyses data set. The table displays the phenotype, obesity risk effect allele, the frequency of the effect allele, the estimated additive effect and the related nominal p-value are derived from publicly available resources. The effect is displayed using the measurement regarded most appropriate for the design of the GWAS. The remaining columns correspond to the respective results observed GWAS meta-analysis. (0.54 MB DOC) [file pgen.1000916.s010.doc]

**Table S4.** DISCOVERY: GWAS-based SNPs of previously reported candidate markers for BMI and/or obesity sorted by chromosome and physical position. The first two columns indicate the name of a previously identified marker and the implied, described candidate genes (in bold those which were confirmed and which are reported in the introduction of the main text). The columns 6-11 summarize the data of three recently published large-scale GWAS (Willer et al., 2009 (publication “WI” and “WI.b” for the Appendix of “WI.b”), Thorleifsson et al., 2009 (publication “TH”), and Meyre et al., 2009 (publication “ME”)^a^). Note that parts of the data sets in Meyre et al. (2009) overlap with our meta-analyses data set. The table displays the phenotype, obesity risk effect allele, the frequency of the effect allele, the estimated additive effect and the related nominal p-value are derived from publicly available resources. The effect is displayed using the measurement regarded most appropriate for the design of the GWAS. The remaining columns correspond to the respective results observed GWAS meta-analysis.

| implied/nearby GENE | SNP | proxy | chromosome | physical position |  | recent GWAS-based candidate markers  (Willer et al., 2009, Thorleifsson et al., 2009 and Meyre et al., 2009^a^) | | | | | |  | GWAS meta-analysis for early onset extreme obesity | | |
| --- | --- | --- | --- | --- | --- | --- | --- | --- | --- | --- | --- | --- | --- | --- | --- |
|  |  |  |  |  |  | publication^a^ | phenotype | obesity risk effect allele^b^ | frequency^c^ | effect (beta or odds ratio) | p-value |  | effect (odds ratio German GWAS) | effect (odds ratio French GWAS) | combined p-value |
| *--* | rs3766431 | -- | 1 | 54502624 |  | TH | BMI/weight | A | 0.446/0.427/0.415/0.423 | 0.17 | 1.6x10^-5^ |  | 0.81 | 1.05 | 1.000 |
| ***NEGR1*** | rs1776012 | -- | 1 | 72490331 |  | TH | BMI/weight | A | 0.455/0.469/0.525/0.311 | 0.17 | 8.9x10^-6^ |  | 1.31 | 1.11 | 0.006 |
| ***NEGR1*** | rs9424977 | -- | 1 | 72497932 |  | TH | BMI/weight | T | 0.458/0.468/0.526/0.454 | 0.17 | 4.6x10^-6^ |  | 1.33 | 1.12 | 0.005 |
| ***NEGR1*** | rs3101336 | -- | 1 | 72523773 |  | TH | BMI/weight | C | 0.582/0.592/0.634/0.528 | 0.21 | 1.1x10^-7^ |  | 1.20 | 1.08 | 0.072 |
| ***NEGR1*** | rs2568958 | -- | 1 | 72537704 |  | TH | BMI/weight | A | 0.581/0.587/0.634/0.528 | 0.21 | 9.9x10^-8^ |  | 1.20 | 1.08 | 0.072 |
| ***NEGR1*** | rs2815752 | -- | 1 | 72585028 |  | WI | BMI | A | 0.620 | 0.1 | 6.0x10^–8^ |  | 1.23 | 1.06 | 0.065 |
| *--* | rs1973993 | -- | 1 | 96716582 |  | TH | BMI/weight | C | 0.625/0.598/0.593/0.254 | 0.17 | 1.3x10^-5^ |  | 1.11 | 1.18 | 0.037 |
| *--* | rs10783050 | -- | 1 | 96809671 |  | TH | BMI/weight | C | 0.358/0.357/0.356/0.083 | 0.19 | 2.8x10^-6^ |  | 1.17 | 1.03 | 0.228 |
| ***SEC16B*** | rs10913469 | -- | 1 | 176180142 |  | TH | BMI/weight | C | 0.200/0.199/0.203/0.255 | 0.19 | 4.2x10^-6^ |  | 1.39 | 1.20 | 0.005 |
| *SIPA1L2* | rs10910555 | *rs12127438* | 1 | 230427413 |  | WI.b | BMI | T*(A)* | 0.650 | -- | 1.3x10^-2^ |  | *0.90* | *0.96* | *0.342* |
| ***TMEM18*** | rs2867125 | -- | 2 | 612827 |  | TH | BMI/weight | C | 0.836/0.834/0.814/0.891 | 0.31 | 1.1x10^-10^ |  | 1.52 | 1.27 | 0.001 |
| ***TMEM18*** | rs6548238 | *rs2947411* | 2 | 624905 |  | WI | BMI | C*(A)* | 0.840 | 0.26 | 1.4x10^-18^ |  | *1.61* | *1.32* | *5.11x10^-5^* |
| ***TMEM18*** | rs4854344 | -- | 2 | 628144 |  | TH | BMI/weight | T | 0.839/0.825/0.815/0.777 | 0.28 | 2.9x10^-10^ |  | 1.47 | 1.33 | 1.52x10^-4^ |
| ***TMEM18*** | rs7561317 | -- | 2 | 634953 |  | TH | BMI/weight | G | 0.837/0.832/0.814/0.760 | 0.29 | 2.4x10^-10^ |  | 1.47 | 1.32 | 2.06x10^-4^ |
| *KBTBD9* | rs1709339 | -- | 2 | 23637258 |  | ME | obesity | C | 0.701 | 1.39 | 2.0x10^-5^ |  | 1.32 | 1.08 | 0.038 |
| *CRIM1* | rs1439845 | -- | 2 | 35264929 |  | WI.b | BMI | G | 0.630 | -- | 2.0x10^-4^ |  | 1.14 | 1.06 | 0.197 |
| *PRKCE* | rs10206343 | -- | 2 | 46019476 |  | WI.b | BMI | T | 0.870 | -- | 1.3x10^-2^ |  | -- | -- | -- |
| *KIAA1155* | rs413693 | -- | 2 | 71257124 |  | ME | obesity | G | 0.124 | 1.47 | 2.0x10^-6^ |  | 0.98 | 1.21 | 0.217 |
| *LOC646295* | rs299575 | -- | 2 | 123525431 |  | ME | obesity | G | 0.630 | 1.47 | 1.0x10^-5^ |  | 0.86 | 1.45 | 0.015 |
| *FLJ34870* | rs16829231 | *rs13410393* | 2 | 134248092 |  | ME | obesity | T*(T)* | 0.825 | 1.69 | 8.0x10^-6^ |  | *1.10* | *1.11* | *0.251* |
| *CXCR4* | rs2011946 | -- | 2 | 136534086 |  | ME | obesity | T | 0.514 | 1.47 | 2.0x10^-6^ |  | 1.18 | 1.43 | 1.36x10^-5^ |
| *BHLHB2* | rs908078 | -- | 3 | 4999771 |  | ME | obesity | C | 0.137 | 1.33 | 4.0x10^-6^ |  | 0.95 | 1.41 | 0.040 |
| *LOC643272* | rs6796959 | -- | 3 | 6262176 |  | ME | obesity | C | 0.506 | 1.41 | 8.0x10^-6^ |  | 1.02 | 0.94 | 0.692 |
| *FNDC3B* | rs12633433 | -- | 3 | 173385769 |  | ME | obesity | G | 0.856 | 1.16 | 9.0x10^-6^ |  | -- | -- | -- |
| ***ETV5*** | rs7647305 | -- | 3 | 187316984 |  | TH | BMI/weight | C | 0.772/0.803/0.769/0.622 | 0.21 | 3.1x10^-6^ |  | 1.18 | 1.02 | 0.320 |
| *KCTD8* | rs752238 | -- | 4 | 44184606 |  | WI.b | BMI | G | 0.770 | -- | 2.5x10^-4^ |  | 0.88 | 1.01 | 1.000 |
| ***GNPDA2*** | rs10938397 | *rs13130484* | 4 | 44877284 |  | WI | BMI | G*(T)* | 0.450 | 0.19 | 3.4x10^-16^ |  | *1.29* | *1.11* | *0.011* |
| ***GNPDA2*** | rs10938397 | *rs13130484* | 4 | 44877284 |  | WI.b | BMI | G*(T)* | 0.480 | -- | 7.5x10^-13^ |  | *1.29* | *1.11* | *0.011* |
| *ODZ3* | rs2726814 | -- | 4 | 183378098 |  | WI.b | BMI | T | 0.110 | -- | 2.3x10^-4^ |  | -- | -- | -- |
| *CDH10* | rs17465346 | -- | 5 | 25486507 |  | WI.b | BMI | T | 0.950 | -- | 6.8x10^-3^ |  | 1.23 | 0.89 | 1.000 |
| *--* | rs467650 | *rs469930* | 5 | 97997353 |  | TH | BMI/weight | T*(A)* | 0.700/0.670/0.666/0.354 | 0.19 | 4.2x10^-6^ |  | *0.88* | *1.06* | *1.000* |
| *--* | rs1047440 | -- | 5 | 122709733 |  | TH | BMI/weight | C | 0.570/0.560/0.563/0.667 | 0.11 | 0.00084 |  | 0.92 | 1.03 | 1.000 |
| *--* | rs2115172 | -- | 5 | 122713027 |  | TH | BMI/weight | A | 0.565/0.556/0.562/0.653 | 0.11 | 0.00097 |  | 0.92 | 1.04 | 1.000 |
| *LOC153328* | rs7717673 | -- | 5 | 135239365 |  | ME | obesity | T | 0.081 | 1.56 | 6.0x10^-6^ |  | 0.92 | 1.64 | 0.013 |
| *COL23A1* | rs11956401 | -- | 5 | 177780715 |  | ME | obesity | A | 0.125 | 1.46 | 2.0x10^-6^ |  | 1.02 | 1.29 | 0.078 |
| *PRL* | rs4712652 | -- | 6 | 22186594 |  | ME | obesity | A | 0.501 | 1.32 | 1.0x10^-6^ |  | 1.04 | 1.23 | 0.025 |
| ***AIF1*** | rs2844479 | *rs805297* | 6 | 31680935 |  | TH | BMI/weight | A*(G)* | 0.676/0.660/0.630/0.686 | 0.16 | 6.1x10^-5^ |  | *1.06* | *0.99* | *1.000* |
| ***AIF1*** | rs2260000 | -- | 6 | 31701455 |  | TH | BMI/weight | A | 0.672/0.632/0.614/0.912 | 0.12 | 0.00046 |  | -- | -- | -- |
| ***AIF1*** | rs1077393 | -- | 6 | 31718508 |  | TH | BMI/weight | A | 0.563/0.518/0.491/0.657 | 0.13 | 0.00047 |  | -- | -- | -- |
| *HLA-DQA2* | rs9275582 | -- | 6 | 32788048 |  | ME | obesity | T | 0.161 | 1.60 | 5.0x10^-6^ |  | 0.88 | 1.55 | 0.010 |
| *MOCS1* | rs12210863 | *rs10807218* | 6 | 40182179 |  | WI.b | BMI | G*(C)* | 0.860 | -- | 3.4x10^-2^ |  | *1.04* | *1.15* | *0.310* |
| *DSCR1L1* | rs6911147 | *rs11963572* | 6 | 46415587 |  | WI.b | BMI | T*(C)* | 0.620 | -- | 6.9x10^-5^ |  | *1.07* | *1.10* | *0.198* |
| *C6ofr143* | rs6907460 | -- | 6 | 54989006 |  | WI.b | BMI | A | 0.390 | -- | 8.3x10^-4^ |  | 0.84 | 1.07 | 1.000 |
| *C6ofrf57* | rs9455181 | -- | 6 | 71359438 |  | WI.b | BMI | T | 0.230 | -- | 4.0x10^-3^ |  | -- | -- | -- |
| *RP5-875H10.1* | rs646839 | -- | 6 | 148299726 |  | ME | obesity | G | 0.779 | 1.64 | 4.0x10^-7^ |  | 1.06 | 1.61 | 1.29x10^-4^ |
| *LOC340268* | rs6463923 | -- | 7 | 9537527 |  | ME | obesity | A | 0.297 | 1.52 | 1.0x10^-6^ |  | -- | -- | -- |
| *TMEM195* | rs2908338 | -- | 7 | 15176204 |  | ME | obesity | G | 0.160 | 1.44 | 4.0x10^-7^ |  | 0.90 | 1.40 | 0.060 |
| *C7ofr9* | rs16873846 | -- | 7 | 25348456 |  | WI.b | BMI | T | 0.950 | -- | 1.2x10^-2^ |  | -- | -- | -- |
| *CALCR* | rs2158044 | -- | 7 | 93006335 |  | ME | obesity | G | 0.452 | 1.43 | 5.0x10^-6^ |  | 0.95 | 1.02 | 1.000 |
| *KLF14* | rs11976955 | -- | 7 | 130084134 |  | WI.b | BMI | C | 0.690 | -- | 1.7x10^-4^ |  | 0.94 | 0.92 | 0.277 |
| *--* | rs17069257 | -- | 8 | 4030923 |  | TH | BMI/weight | C | 0.133/0.170/0.164/0.212 | 0.18 | 3.6x10^-5^ |  | 0.99 | 1.10 | 0.590 |
| *SFRP1* | rs11996523 | -- | 8 | 41115317 |  | ME | obesity | G | 0.778 | 1.52 | 1.0x10^-5^ |  | 1.16 | 0.92 | 1.000 |
| *LOC138046* | rs11773921 | -- | 8 | 84787572 |  | WI.b | BMI | A | 0.140 | -- | 5.3x10^-4^ |  | -- | -- | -- |
| *KIAA1429* | rs3102841 | -- | 8 | 95588381 |  | ME | obesity | T | 0.447 | 1.36 | 1.0x10^-7^ |  | 1.03 | 1.04 | 0.577 |
| *FLJ45872* | rs1858367 | -- | 8 | 138999961 |  | ME | obesity | G | 0.036 | 2.98 | 2.0x10^-10^ |  | 0.99 | 0.98 | 0.868 |
| *PTCH1* | rs4743120 | -- | 9 | 97437280 |  | WI.b | BMI | T | 0.350 | -- | 5.8x10^-4^ |  | -- | -- | -- |
| *--* | rs6477693 | -- | 9 | 110958186 |  | TH | BMI/weight | C | 0.308/0.273/0.260/0.281 | 0.14 | 0.00015 |  | 1.04 | 1.00 | 1.000 |
| *ASTN2* | rs4091697 | -- | 9 | 118694342 |  | ME | obesity | T | 0.083 | 1.51 | 2.0x10^-5^ |  | 1.16 | 1.52 | 0.005 |
| *--* | rs867559 | -- | 9 | 128505146 |  | TH | BMI/weight | G | 0.217/0.182/0.195/0.326 | 0.23 | 7.0x10^-6^ |  | 1.14 | 1.06 | 0.307 |
| *ITIH5* | rs4623795 | -- | 10 | 7691915 |  | WI.b | BMI | G | 0.840 | -- | 1.7x10^-2^ |  | 1.11 | 0.85 | 0.074 |
| ***PTER*** | rs10508503 | -- | 10 | 16339957 |  | ME | obesity | C | 0.870 | 2.08 | 1.0x10^-7^ |  | 1.02 | 1.30 | 0.140 |
| *C10orf112* | rs1326986 | *rs12359487* | 10 | 19969519 |  | ME | obesity | G*(G)* | 0.021 | 2.93 | 2.0x10^-7^ |  | *1.37* | *0.93* | *1.000* |
| *RET* | rs3026762 | -- | 10 | 42930677 |  | ME | obesity | T | 0.043 | 2.20 | 6.0x10^-7^ |  | -- | -- | -- |
| *C10orf53* | rs1343772 | -- | 10 | 50554954 |  | ME | obesity | C | 0.408 | 1.42 | 3.0x10^-6^ |  | 0.95 | 0.99 | 0.699 |
| *STK33* | rs10769908 | -- | 11 | 8440665 |  | WI.b | BMI | C | 0.520 | -- | 4.6x10^-7^ |  | 0.93 | 1.11 | 0.572 |
| ***BDNF*** | rs7481311 | -- | 11 | 27539705 |  | TH | BMI/weight | T | 0.237/0.233/0.233/0.305 | 0.23 | 4.9x10^-6^ |  | 1.24 | 1.13 | 0.059 |
| ***BDNF*** | rs4074134 | -- | 11 | 27603861 |  | TH | BMI/weight | C | 0.844/0.789/0.804/0.825 | 0.26 | 1.8x10^-6^ |  | 1.27 | 1.18 | 0.017 |
| ***BDNF*** | rs4923461 | -- | 11 | 27613486 |  | TH | BMI/weight | A | 0.845/0.788/0.804/0.822 | 0.27 | 1.1x10^-6^ |  | 1.27 | 1.16 | 0.020 |
| ***BDNF*** | rs925946 | -- | 11 | 27623778 |  | TH | BMI/weight | T | 0.335/0.295/0.305/0.254 | 0.22 | 2.0x10^-7^ |  | 1.24 | 1.22 | 0.005 |
| ***BDNF*** | rs10501087 | -- | 11 | 27626684 |  | TH | BMI/weight | T | 0.844/0.789/0.806/0.914 | 0.26 | 4.2x10^-6^ |  | 1.32 | 1.16 | 0.014 |
| ***BDNF*** | rs6265 | -- | 11 | 27636492 |  | TH | BMI/weight | C | 0.853/0.802/0.825/0.941 | 0.26 | 7.2x10^-6^ |  | 1.33 | 1.12 | 0.030 |
| ***BDNF*** | rs10835211 | -- | 11 | 27657941 |  | TH | BMI/weight | A | 0.264/0.251/0.253/0.114 | 0.18 | 1.5x10^-5^ |  | 1.19 | 1.18 | 0.031 |
| ***MTCH2*** | rs4752856 | -- | 11 | 47604618 |  | WI.b | BMI | A | 0.340 | -- | 4.7x10^-9^ |  | 1.11 | 0.91 | 0.754 |
| ***MTCH2*** | rs10838738 | -- | 11 | 47619625 |  | WI | BMI | G | 0.340 | 0.07 | 4.6x10^–9^ |  | 1.14 | 0.90 | 0.864 |
| *LOC399950* | rs7940211 | -- | 11 | 111964161 |  | ME | obesity | G | 0.474 | 1.43 | 1.0x10^-5^ |  | -- | -- | -- |
| ***BCDIN3D*** | rs7138803 | -- | 12 | 48533735 |  | TH | BMI/weight | A | 0.373/0.376/0.375/0.184 | 0.22 | 9.6x10^-7^ |  | 1.03 | 1.41 | 0.001 |
| ***BCDIN3D*** | rs836964 | -- | 12 | 48556162 |  | TH | BMI/weight | A | 0.893/0.910/0.882/0.756 | 0.28 | 2.2x10^-6^ |  | 1.43 | 1.28 | 0.006 |
| *LOC643275* | rs6580742 | -- | 12 | 49014078 |  | ME | obesity | C | 0.796 | 1.67 | 4.0x10^-6^ |  | 1.39 | 0.94 | 1.000 |
| *SLC16A7* | rs275982 | -- | 12 | 58213837 |  | WI.b | BMI | A | 0.230 | -- | 1.1x10^-4^ |  | -- | -- | -- |
| *MLXIP* | rs925460 | -- | 12 | 121199956 |  | WI.b | BMI | C | 0.780 | -- | 1.3x10^-4^ |  | 1.13 | 0.96 | 1.000 |
| *--* | rs7336332 | -- | 13 | 26956404 |  | TH | BMI/weight | G | 0.154/0.160/0.156/0.269 | 0.23 | 6.3x10^-6^ |  | 1.14 | 1.12 | 0.199 |
| *SMOC1* | rs227416 | -- | 14 | 69530362 |  | ME | obesity | C | 0.549 | 1.30 | 1.0x10^-5^ |  | 1.01 | 1.37 | 0.003 |
| *NRXN3* | rs1396618 | -- | 14 | 78361657 |  | ME | obesity | C | 0.089 | 1.50 | 1.0x10^-6^ |  | -- | -- | -- |
| *EVL* | rs12588659 | -- | 14 | 99657566 |  | ME | obesity | C | 0.891 | 2.22 | 6.0x10^-8^ |  | 0.99 | 2.33 | 2.26x10^-5^ |
| *MAP1A* | rs2245715 | -- | 15 | 41605344 |  | WI.b | BMI | G | 0.920 | -- | 8.7x10^-4^ |  | 0.99 | 1.09 | 0.660 |
| *SMAD3* | rs11071927 | -- | 15 | 65048226 |  | ME | obesity | C | 0.107 | 1.54 | 1.0x10^-6^ |  | 1.19 | 1.18 | 0.729 |
| *RKHD3* | rs12324805 | -- | 15 | 80139255 |  | WI.b | BMI | C | 0.310 | -- | 6.9x10^-6^ |  | 0.86 | 0.95 | 1.000 |
| *RGMA* | rs7181095 | -- | 15 | 91375823 |  | WI.b | BMI | T | 0.360 | -- | 4.0x10^-5^ |  | -- | -- | -- |
| *IGF1R* | rs8024593 | -- | 15 | 96988019 |  | WI.b | BMI | G | 0.060 | -- | 1.2x10^-3^ |  | -- | -- | -- |
| *A2BP1* | rs4786847 | -- | 16 | 6250234 |  | ME | obesity | G | 0.108 | 1.98 | 9.0x10^-10^ |  | 1.33 | 0.90 | 1.000 |
| ***SH2B1*** | rs8049439 | -- | 16 | 28745016 |  | TH | BMI/weight | C | 0.437/0.400/0.388/0.425 | 0.18 | 6.0x10^-6^ |  | 1.23 | 1.02 | 0.177 |
| ***SH2B1*** | rs4788102 | -- | 16 | 28780899 |  | TH | BMI/weight | A | 0.436/0.390/0.386/0.261 | 0.18 | 3.5x10^-6^ |  | 1.22 | 1.02 | 0.180 |
| ***SH2B1*** | rs7498665 | -- | 16 | 28790742 |  | TH | BMI/weight | G | 0.438/0.390/0.390/0.263 | 0.19 | 1.7x10^-6^ |  | 1.22 | 1.03 | 0.170 |
| ***SH2B1*** | rs7498665 | -- | 16 | 28790742 |  | WI | BMI | G | 0.410 | 0.15 | 5.1x10^-11^ |  | 1.22 | 1.03 | 0.170 |
| ***SH2B1*** | rs9931989 | *rs6565259* | 16 | 28813585 |  | WI.b | BMI | G*(C)* | 0.410 | -- | 4.9x10^-11^ |  | *1.18* | *1.01* | *0.281* |
| ***FTO*** | rs6499640 | -- | 16 | 52327178 |  | TH | BMI/weight | A | 0.647/0.613/0.599/0.635 | 0.25 | 6.0x10^-8^ |  | 1.15 | 1.11 | 0.069 |
| ***FTO*** | rs1421085 | -- | 16 | 52358455 |  | ME | obesity | -- | -- | -- | 3.0x10^-12^ |  | 1.58 | 1.33 | 2.98x10^-8^ |
| ***FTO*** | rs1421085 | -- | 16 | 52358455 |  | WI.b | BMI | C | 0.410 | -- | 9.5x10^-52^ |  | 1.58 | 1.33 | 2.99 x10^-8^ |
| ***FTO*** | rs8050136 | -- | 16 | 52373776 |  | TH | BMI/weight | A | 0.408/0.387/0.413/0.427 | 0.37 | 4.4x10^-24^ |  | 1.52 | 1.35 | 8.96 x10^-8^ |
| ***FTO*** | rs3751812 | -- | 16 | 52375961 |  | TH | BMI/weight | T | 0.407/0.387/0.410/0.106 | 0.4 | 3.3x10^-24^ |  | 1.53 | 1.34 | 9.03 x10^-8^ |
| ***FTO*** | rs9939609 | -- | 16 | 52378028 |  | WI | BMI | A | 0.410 | 0.33 | 4.3x10^–51^ |  | 1.52 | 1.35 | 8.88 x10^-8^ |
| ***FTO*** | rs7190492 | -- | 16 | 52386253 |  | TH | BMI/weight | G | 0.621/0.587/0.655/0.779 | 0.31 | 2.0x10^-12^ |  | 1.28 | 1.11 | 0.017 |
| ***FTO*** | rs8044769 | -- | 16 | 52396636 |  | TH | BMI/weight | C | 0.518/0.506/0.544/0.759 | 0.31 | 7.9x10^-16^ |  | 1.30 | 1.18 | 0.002 |
| ***MAF*** | rs1424233 | -- | 16 | 78240252 |  | ME | obesity | A | 0.434 | 1.39 | 1.0x10^-5^ |  | 0.95 | 0.99 | 0.702 |
| *DKFZp434O0320* | rs987052 | -- | 16 | 84879960 |  | ME | obesity | G | 0.101 | 1.79 | 1.0x10^-7^ |  | 1.40 | 1.10 | 0.044 |
| *NTN1* | rs11078773 | -- | 17 | 8845288 |  | WI.b | BMI | C | 0.510 | -- | 9.7x10^-4^ |  | 1.07 | 1.04 | 0.479 |
| ***NPC1*** | rs1805081 | -- | 18 | 19394430 |  | ME | obesity | A | 0.576 | 1.47 | 2.0x10^-6^ |  | 1.01 | 0.85 | 0.172 |
| *SEC11L3* | rs1380100 | -- | 18 | 54929690 |  | ME | obesity | T | 0.099 | 1.50 | 4.0x10^-6^ |  | 0.83 | 1.48 | 0.120 |
| ***MC4R*** | rs17700144 | -- | 18 | 55962962 |  | WI.b | BMI | A | 0.220 | -- | 9.8x10^-19^ |  | 1.50 | 1.48 | 2.40x10^-8^ |
| ***MC4R*** | rs633265 | -- | 18 | 55982448 |  | TH | BMI/weight | T | 0.464/0.463/0.425/0.771 | 0.14 | 8.9x10^-5^ |  | 1.35 | 1.42 | 4.68x10^-7^ |
| ***MC4R*** | rs1350341 | -- | 18 | 55993513 |  | TH | BMI/weight | A | 0.461/0.462/0.424/0.769 | 0.14 | 6.4x10^-5^ |  | 1.35 | 1.41 | 6.90x10^-7^ |
| ***MC4R*** | rs17782313 | -- | 18 | 56002077 |  | ME | obesity | -- | -- | -- | 3.0x10^-7^ |  | 1.47 | 1.45 | 2.28x10^-7^ |
| ***MC4R*** | rs17782313 | -- | 18 | 56002077 |  | WI | BMI | C | 0.210 | 0.2 | 4.6x10^-18^ |  | 1.47 | 1.45 | 2.28x10^-7^ |
| ***MC4R*** | rs12970134 | -- | 18 | 56035730 |  | TH | BMI/weight | A | 0.299/0.282/0.257/0.159 | 0.19 | 2.6x10^-6^ |  | 1.38 | 1.36 | 1.11x10^-5^ |
| *LOC400657* | rs7506051 | -- | 18 | 70407597 |  | ME | obesity | A | 0.134 | 1.71 | 7.0x10^-7^ |  | -- | -- | -- |
| *MUC16* | rs1423052 | *rs11882256* | 19 | 8922559 |  | WI.b | BMI | G*(T)* | 0.260 | -- | 2.8x10^-3^ |  | *0.90* | *1.13* | *0.620* |
| ***KCTD15*** | rs29941 | -- | 19 | 39001372 |  | TH | BMI/weight | G | 0.691/0.682/0.684/0.791 | 0.19 | 5.6x10^-6^ |  | 1.23 | 1.06 | 0.082 |
| ***KCTD15*** | rs11084753 | -- | 19 | 39013977 |  | WI | BMI | G | 0.670 | 0.06 | 2.3x10^-8^ |  | 1.33 | 1.08 | 0.018 |
| ***KCTD15*** | rs11084753 | -- | 19 | 39013977 |  | WI.b | BMI | G | 0.670 | -- | 2.3x10^-8^ |  | 1.33 | 1.08 | 0.018 |
| *BMP2* | rs2145270 | -- | 20 | 6569685 |  | WI.b | BMI | T | 0.650 | -- | 7.2x10^-6^ |  | 1.23 | 0.98 | 1.000 |
| *TASP1* | rs636887 | *rs1076052* | 20 | 13235035 |  | WI.b | BMI | C*(T)* | 0.450 | -- | 1.4x10^-3^ |  | *0.87* | *1.14* | *0.659* |
| *LOC391258* | rs6100226 | -- | 20 | 56807638 |  | ME | obesity | C | 0.681 | 1.45 | 2.0x10^-5^ |  | 0.83 | 1.39 | 0.059 |
| *FAM19A5* | rs4823535 | *rs4823531* | 22 | 47209622 |  | WI.b | BMI | G*(G)* | 0.690 | -- | 1.6x10^-1^ |  | *1.20* | *1.05* | *0.133* |

^a^ note that parts of the data from Meyre et al. (2009) have been re-analyzed here

^b^ as reported in the publications

^c^ for Thorleifsson et al. (2009) data on three populations were reported in the Appendix of Thorleifsson et al. (2009)
