# Supplementary material for: Two New Loci for Body-Weight Regulation Identified in a Joint Analysis of Genome-Wide Association Studies for Early-Onset Extreme Obesity in French and German Study Groups
Source: PLoS Genet. 2010 Apr 22;6(4):e1000916. doi: 10.1371/journal.pgen.1000916 (PMC2858696; doi:10.1371/journal.pgen.1000916)
Supplement: Table S5 — DISCOVERY: SNPs of previously identified ‘classical’ obesity candidate genes. The first column indicates the name of a previously identified candidate gene. The second column indicates SNPs which showed strongest association in Scuteri et al. (2007) for the phenotype, effect allele, frequency, the estimated additive effect and the related nominal p-value in columns 6–9. The remaining columns correspond to the respective results observed in our GWAS meta-analysis (only markers with two-sided adjusted p-values <.1 and the ‘directionally consistent’ obesity risk effect allele are displayed). (0.07 MB DOC) [file pgen.1000916.s011.doc]

**Table S5.** DISCOVERY: SNPs of previously identified ‘classical’ obesity candidate genes. The first column indicates the name of a previously identified candidate gene. The second column indicates SNPs which showed strongest association in Scuteri et al. (2007) for the phenotype, effect allele, frequency, the estimated additive effect and the related nominal p-value in columns 6-9. The remaining columns correspond to the respective results observed in our GWAS meta-analysis (only markers with two-sided adjusted p-values <.1 and the 'directionally consistent' obesity risk effect allele are displayed).

| gene | SNP | chromosome | physical position | obesity risk effect alleles | Scuteri et al. (2007) | | | |  | GWAS meta-analysis for early onset extreme obesity | | |
| --- | --- | --- | --- | --- | --- | --- | --- | --- | --- | --- | --- | --- |
|  |  |  |  |  | phenotype | freq | effect (beta) | p-value |  | effect (odds ratio German GWAS) | effect (odds ratio French GWAS) | combined  p-value |
| *BDNF* | rs12288512 | 11 | 27704247 | A | BMI | 0.900 | 0.057 | 0.186 |  | 1.11 | 1.32 | 0.013 |
| *BDNF* | rs12288512 | 11 | 27704247 | A | weight | 0.900 | 0.054 | 0.160 |  | 1.11 | 1.32 | 0.013 |
| *UCP3* | rs11235972 | 11 | 73394722 | G | weight | 0.870 | 0.041 | 0.240 |  | 1.39 | 1.09 | 0.020 |
| *UCP3* | rs11235972 | 11 | 73394722 | G | BMI | 0.870 | 0.043 | 0.353 |  | 1.39 | 1.09 | 0.020 |
| *NMB* | rs1107179 | 15 | 82999366 | T | hip | 0.590 | 0.033 | 0.260 |  | 1.03 | 1.250 | 0.023 |
| *NMB* | rs1107179 | 15 | 82999366 | T | BMI | 0.590 | 0.015 | 0.636 |  | 1.03 | 1.25 | 0.023 |
| *NMB* | rs1107179 | 15 | 82999366 | T | weight | 0.590 | 0.013 | 0.611 |  | 1.03 | 1.25 | 0.023 |
| *LPL^a^* | rs17410783 (rs10108800) | 8 | 19868351 | G | hip | 0.820 | 0.105 | 0.006 |  | 1.01 | 1.28 | 0.045 |
| *LPL^a^* | rs17410783 (rs10108800) | 8 | 19868351 | G | BMI | 0.820 | 0.087 | 0.014 |  | 1.01 | 1.28 | 0.045 |
| *LPL^a^* | rs17410783 (rs10108800) | 8 | 19868351 | G | weight | 0.820 | 0.076 | 0.017 |  | 1.01 | 1.28 | 0.045 |
| *CRP* | rs876538 | 1 | 157942341 | T | hip | 0.870 | 0.089 | 0.037 |  | 1.10 | 1.19 | 0.064 |
| *CRP* | rs876538 | 1 | 157942341 | T | BMI | 0.870 | 0.077 | 0.048 |  | 1.10 | 1.19 | 0.064 |
| *CRP* | rs876538 | 1 | 157942341 | T | weight | 0.870 | 0.073 | 0.042 |  | 1.10 | 1.19 | 0.064 |

^a^ for rs17410783 the proxy marker rs10108800 was used; recently rs17410783 has been merged into rs3200218
