# Supplementary material for: Two New Loci for Body-Weight Regulation Identified in a Joint Analysis of Genome-Wide Association Studies for Early-Onset Extreme Obesity in French and German Study Groups
Source: PLoS Genet. 2010 Apr 22;6(4):e1000916. doi: 10.1371/journal.pgen.1000916 (PMC2858696; doi:10.1371/journal.pgen.1000916)
Supplement: Text S1 — DISCOVERY and GENERALIZATION. (0.27 MB DOC) [file pgen.1000916.s012.doc]

**Supporting Information
for**

**„Two new loci for body-weight regulation identified in a joint analysis of genome-wide association studies for early onset extreme obesity**

**in French and German study groups”**

by

**André Scherag, Christian Dina, Anke Hinney, Vincent Vatin, Susann Scherag, Carla I. G. Vogel, Timo D. Müller, Harald Grallert, H-Erich Wichmann, Beverley Balkau, Barbara Heude, Marjo-Riitta Jarvelin, Anna-Liisa Hartikainen, Claire Levy-Marchal, Jacques Weill, Jérôme Delplanque, Antje Körner, Wieland Kiess, Peter Kovacs, Nigel W. Rayner, Inga Prokopenko, Mark I. McCarthy, Helmut Schäfer, Ivonne Jarick, Heiner Boeing, Eva Fisher, Thomas Reinehr, Joachim Heinrich, Peter Rzehak, Dietrich Berdel, Michael Borte, Heike Biebermann, Heiko Krude, Dieter Rosskopf, Christian Rimmbach, Winfried Rief, Tobias Fromme, Martin Klingenspor, Annette Schürmann, Nadja Schulz, Markus M. Nöthen, Thomas W. Mühleisen, Raimund Erbel , Karl-Heinz Jöckel, Susanne Moebus, Tanja Boes, Thomas Illig, Philippe Froguel, Johannes Hebebrand, and David Meyre**

[1 DISCOVERY 2](#__RefHeading___Toc255907073)

[1.1 Genome-wide association studies for early onset extreme obesity 2](#__RefHeading___Toc255907074)

[1.1.1 French GWAS 2](#__RefHeading___Toc255907075)

[1.1.2 German GWAS 2](#__RefHeading___Toc255907076)

[1.1.3 Imputations, combined analyses and marker selection 3](#__RefHeading___Toc255907077)

[1.1.4 Power considerations for the GWAS 4](#__RefHeading___Toc255907078)

[1.1.5 Candidate gene analyses 6](#__RefHeading___Toc255907079)

[1.2 Follow-up of GWAS signals for early onset extreme obesity 8](#__RefHeading___Toc255907080)

[1.2.1 Case-control studies with obese Children and adolescents of European origin 8](#__RefHeading___Toc255907081)

[1.2.2 Family-based association studies with extremely obese children and adolescent case-control studies of European origin 8](#__RefHeading___Toc255907082)

[1.2.3 Marker selection for GENERALIZATION 9](#__RefHeading___Toc255907083)

[1.2.4 Power considerations for the Follow-up 9](#__RefHeading___Toc255907084)

[2 GENERALIZATION 11](#__RefHeading___Toc255907085)

[2.1 Children and adolescents of European origin 11](#__RefHeading___Toc255907086)

[2.1.1 Obesity sample: Datteln Paediatric Obesity sample (T. Reinehr) 11](#__RefHeading___Toc255907087)

[2.1.2 Population-based samples: GINI/LISA (J. Heinrich, P. Rzehak, D. Berdel, M. Borte) and Berlin School Girls sample (H. Krude, H. Biebermann) 11](#__RefHeading___Toc255907088)

[2.2 Adults of European origin 12](#__RefHeading___Toc255907089)

[2.2.1 Obesity sample: Marburg Adult Obesity sample (W. Rief) 12](#__RefHeading___Toc255907090)

[2.2.2 Population-based sample: The EPIC-Potsdam Study (H. Boeing, E. Fisher) 12](#__RefHeading___Toc255907091)

[2.2.3 Population-based sample: KORA (H.E. Wichmann, T. Illig, H. Grallert) 13](#__RefHeading___Toc255907092)

[2.2.4 Population-based sample: SHIP (D. Rosskopf, C. Rimmbach) 13](#__RefHeading___Toc255907093)

[2.2.5 Population-based sample: The Heinz-Nixdorf Recall Study (K.H. Jöckel, R. Erbel, S. Moebus) 14](#__RefHeading___Toc255907094)

# DISCOVERY

- 1. Genome-wide association studies for early onset extreme obesity
     1. French GWAS

#### Participants

663 French obese children, recruited by the CNRS UMR8090 and another 22 obese children who were patients of Toulouse Children’s Hospital were genotyped as ‘cases’ (Table S1) along with 655 normal-weight French children selected from the STANISLAS study [1] and 30 lean children from the Fleurbaix-Laventie Ville Santé II study [2] (‘controls’, Table S1). More details on the samples can be found in Meyre et al. [3].

#### Genotyping and low level quality control analyses

For the French GWAS genotyping was performed on the Illumina Human CNV370-Duo array using 750ng of genomic DNA following the manufacturer’s protocols (Illumina Inc., USA). Genotypes were called using Illumina’s BeadStation genotyping solutions, based on the GenCall software application to automatically cluster, call genotypes, and assign confidence scores. The GenCall application incorporates a clustering algorithm (GenTrain). SNPs with a call rate ≤ 95%, evidence for departure from Hardy-Weinberg equilibrium in the control group (exact two-sided p ≤ .001), and with minor allele frequency below 1 % in the total sample were excluded from the final analysis (308,846 SNPs retained; for details see Table S2 and Meyre et al. [3]). The study was approved by the local ethics committees and conducted in accordance with the guidelines of *The Declaration of Helsinki*.

- - 1. German GWAS

#### Participants

435 extremely obese children and adolescents (‘cases’) were recruited in hospitals specialized for the inpatient treatment of extreme obesity while 435 healthy lean individuals (‘controls’) were ascertained at the University of Marburg (Table S1). We relied on older healthy underweight controls to substantially reduce the probability of their becoming overweight and to increase power. In Hinney et al. [4] we also show that control group mainly comprises individuals who presumably also were in the lower body weight range during adolescence. Written informed consent was given by all participants and in case of minors by their parents. The study was approved by the Ethics Committees of the Universities of Marburg and Essen and conducted in accordance with the guidelines of *The Declaration of Helsinki*.

#### Genotyping and low level quality control analyses

For the German GWAS genotyping was performed on the Genome-Wide Human SNP Array 6.0 (<http://www.affymetrix.com/>) at the Affymetrix Services Lab (California, USA). Genotypes were called using the „birdseed calling“-algorithm [5]. Similar to the French GWAS, SNPs with a call rate ≤ 95%, evidence for departure from Hardy-Weinberg equilibrium in the control group (exact two-sided p ≤ .001), and with minor allele frequency below 1 % in the total sample were excluded from the final analysis (730,577 SNPs retained; for details see Table S2 and Hinney et al. [4]).

- - 1. Imputations, combined analyses and marker selection

Both GWAS were conducted on different genome-wide arrays (French GWAS: Illumina Human CNV370-Duo; German GWAS: Affymetrix Genome-Wide Human SNP Array 6.0) with different marker sets. To allow for a combined analysis across different marker sets and to improve coverage of the genome, imputation of polymorphic HapMap CEU SNPs (release 22) was performed using PLINK [6]. To address imputation accuracy issues raised against PLINK [7] we also performed imputations using IMPUTE [8]. These IMPUTE imputations were run by an independent group at the University of Oxford and lead to a very similar order of the strongest association signals. To run the imputations, the alleles of the markers of both GWAS and the map positions were altered to match those of the HapMap CEU (release 22). Markers not on the HapMap CEU (release 22) panel were discharged in order to omit imputation problems. Following the suggestion of the authors [6], only imputed SNPs with an INFO value > .8 in both GWAS were further proceeded which reduced the number of SNPs from 2,239,392 (minor allele frequency greater than 1 % and genotyping rate greater than .95 in the 60 CEU founders) to 1,596,878 autosomal SNPs (1,641,891 SNPs (73%) with available p-values on both GWAS samples including the x chromosome). Afterwards, we decided to combine the two imputed data sets by first deriving one-sided p-values from the chi-square statistic of each SNP. Secondly, we calculated combined one-sided p-values using the weighted inverse normal method with the weights chosen proportional to the size of the samples (.61 for the French GWAS and .39 for the German GWAS). To increase the chances to detect true signals, we initially assumed that the direction of the effects for C/G or A/T SNPs would be the same (for the follow-up of the DISCOVERY step, minor allele frequencies were used to derive the obesity risk effect allele). Conversely, this may come at the price of some inflated test statistics which was taken care of by deflating the respective p-values (Figure 2 and Figure S1 that are both based on two-sided p-values (min(1, 2 x one-sided p-value))). The deflation was done by first generating random test statistics under the global null hypotheses which were processed exactly like the observed test statistics. Secondly, we used robust linear regression (package rlm in R 2.9.0) to compare the ordered observed and generated, theoretical –log_10_(p-values) and took the estimated regression coefficient 1.15 to deflate the observed –log_10_(p-values) thus mimicking genomic control analyses. Note that the genomic control (GC) parameter for each GWAS prior to the imputation were λGC_French=1.11 and λGC_German=1.18. To support the validity of our proceeding, we also performed sensitivity analyses using GC corrected markerwise statistics and applied the software METAL (http://www.sph.umich.edu/csg/abecasis/Metal/index.html) to meta-analyse the two GWAS. These analyses lead to combined p-values that were even more liberal (smaller) than the ones that we derived.

Regions of interest were then derived by requiring that the unadjusted two-sided p-values had to be ≤10^-5^. Applying this filter to the already reduce number of 1,596,878 SNPs leads to 217 SNPs. In this reduced marker panel, we observed 21 regions of interest (213 SNPs distributed as shown in Figure S5) according to the smallest combined p-values with more than one SNP showing some evidence for an association (more than a single SNP within a locus (lead SNP ± 500 kb) showing evidence for association (with a p-value ≤5x10^-4^)). In both GWAS, the intensity plots of all 21 regions were checked for genotype calling errors. Within each region we also selected, where possible, proxy-markers with a similarly low p-value and a similar allele frequency addressing between-marker LD. The list in Table S3 is the result of this proceeding.

- - 1. Power considerations for the GWAS

Power considerations using QUANTO 1.2.3 (<http://hydra.usc.edu/gxe>) revealed that a joint GWAS sample of about 1,000 case-control pairs has a comparison-wise power ≥ .8 to detect a log-additive OR ≥ 2.2 for minor allele frequencies (MAFs) of 1% or a log-additive OR ≥ 1.3 for MAFs of 15% (α=.05; two-sided; rare disease assumption). If multiple testing of 1,596,878 SNPs (see 1.1.3) is taken into account, a Bonferroni-corrected genomewide significance level of α_BF_≈3.1x10^-8^ (two-sided) may be applied. In this case, a joint GWA sample of about 1,000 case-control pairs has a comparison-wise power ~ .8 to detect a log-additive OR ≥ 2.2 for MAFs ≥ 5 %.

To compare this standard case-control design to a population-based approach, we also performed calculations using the Genetic Power Calculator [9] available at <http://pngu.mgh.harvard.edu/~purcell/gpc/>. We determined the comparison-wise power of the case-control approach based on a normally distributed QTL and standardized effect sizes of .05 and .1 in units of standard deviations for each risk allele under an additive mode of inheritance without dominance effects and the same standard deviation of 1 with a MAF of 1%, 5%, 10%, 25% and 40%. Note that a standardized effect of .1 corresponds to the effect observed for rs9939609 of the *FTO* gene [10] for which effects on the odds ratio scale have also been reported (odds ratio for obesity in the adult general population 1.31 per obesity risk effect allele). Cases were defined as having a QTL ≥ 97th percentile or ≥ 99th percentile which corresponds to 1.88 or 2.33 on the z-score scale while controls were defined as between ≥ 25th and < 75th percentile (±.67 on the z-score scale). For the case of direct mapping, we selected 1,000 case-control pairs and applied an arbitrarily chosen significance level of α=.05 (two-sided). Next, we determined the number of population-based individuals required to achieve the same power as in the case-control approach for the same underlying alternative hypothesis and significance level. The calculations (see Table S6 below) show that a design with a more extreme percentile cut-off for cases is more powerful than a design with a less extreme cut-off but has still limited power to detect smaller effects for 1,000 case-control pairs. Most importantly, however, the comparison shows that a case-control design can be more efficient than a population-based approach and that this effect is more pronounced the more extreme the cut-off value is chosen.

**Table S6.** Comparison of power and efficiency of a case-control vs. a population based approach taking selected groups from the population to define cases and controls.

| cut-off for to define cases |  | MAF [%] |  | standardized effect size .05 | |  | standardized effect size .1 | |
| --- | --- | --- | --- | --- | --- | --- | --- | --- |
|  |  |  |  | comparison-wise **power** of 1,000 case-controls pairs [%] | required **number of population- based individuals** for the same power |  | comparison-wise **power** of 1,000 case-controls pairs [%] | required **number of population- based individuals** for the same power |
| 97th percentile |  | 1 |  | 21.29 | 2,992 |  | 69.53 | 3,389 |
|  |  | 5 |  | 19.94 | 2,749 |  | 63.11 | 2,923 |
|  |  | 10 |  | 19.58 | 2,684 |  | 61.22 | 2,797 |
|  |  | 25 |  | 19.22 | 2,620 |  | 59.18 | 2,667 |
|  |  | 40 |  | 19.04 | 2,587 |  | 58.14 | 2,603 |
|  |  |  |  |  |  |  |  |  |
| 99th percentile |  | 1 |  | 28.27 | 4,255 |  | 84.61 | 4,928 |
|  |  | 5 |  | 26.05 | 3,851 |  | 77.94 | 4,137 |
|  |  | 10 |  | 25.48 | 3,748 |  | 75.83 | 3,930 |
|  |  | 25 |  | 24.89 | 3,641 |  | 73.48 | 3,716 |
|  |  | 40 |  | 24.59 | 3,587 |  | 72.26 | 3,611 |

- - 1. Candidate gene analyses

We investigated autosomal candidate markers derived from recently published GWAS [3,11,12]. In addition, we also accessed the same 72 candidate genes previously tested for association with obesity in humans [4,13] and reported by Scuteri et al. [14]: *ACE, ACTN3, ADIPOQ, ADIPOR1, ADIPOR2, ADRB1, ADRB2, AGER, AHSG, APOA2, APOA4, APOA5, BDNF, CASQ1, COL1A1, COMT, CRP, CYP11B2, DIO1, ENPP1, ESR1, ESR2, FABP2, FOXC2, GAD2, GFPT1, GHRHR, GNAS, GNB3, GPR40, H6PD, HSD11B1, ICAM1, IGF1, IGF2, IL6, IL6R, KCNJ11, KL, LEP, LEPR, LIPC, LPL, LTA, MC4R, MCHR1, MKKS, MTHFR, MTTP, NMB, NOS3, NPY, NPY2R, NR0B2, NTRK2, PARD6A, PLIN, PPARG, PPARGC1A, PRDM2, PTPN1, PYY, RETN, SCD, SELE, SERPINE1, TAS2R38, TNF, UCP1, UCP2, UCP3,* and *VDR*. Within each gene of the 72 genes we focussed on the strongest association signal reported by Scuteri et al. [14] to address multiplicity issues to some extent. The results of these analyses can be found in the Tables S4, S5. In the main text, we only focus on the GWAS-based candidate markers. For the classical obesity candidate genes we observed that SNPs in *BDNF, UCP3, NMB, LPL* and *CRP* were moderately associated (p<.1) with the same risk alleles in our jointly analysed GWAS and the GWAS of Scuteri et al. [14] (Table S5).

For the validated markers of the monogenic candidate genes *MC4R* [15,16] and *PCSK1* [17], GWAS data were available for rs6232 (p=.004 with OR in the range of 1.3 to 1.6), rs6234 and rs6235 (both p=.04 with OR in the range of 1.1 to 1.2).

- 1. Follow-up of GWAS signals for early onset extreme obesity
     1. Case-control studies with obese Children and adolescents of European origin

The samples to follow-up the GWAS signals for early-onset obesity included 537 French obese children (320 females; mean age 11.55 ± 3.12 years; mean BMI 29.64 ± 5.70 kg/m²), 114 lean children from the Fleurbaix-Laventie Ville Santé II study [2] and 452 young adult French control subjects (350 females; mean age 21.16 ± 4.38 years; mean BMI 20.81 ± 2.16 kg/m²; mean BMI-SDS -.34 ± .60) from the Haguenau study [18]. This case-control sample is called ‘French’ in Table S3. In addition, 389 obese German children (194 females; mean age 11.43 ± 3.56 years; mean BMI 29.72 ± 5.58 kg/m²; mean BMI-SDS 2.64 ± .54) and 1,135 lean German children (549 females; mean age 11.84 ± 2.87 years; mean BMI 18.60 ± 3.22 kg/m²; mean BMI-SDS .03 ± .93) from Leipzig [19] which are called ‘German’ in Table S3 were genotyped. Finally, extremes (255 obese: 110 females; age 16 years; mean BMI-SDS 2.82 ± .86; 536 lean: 259 females; age 16 years; mean-BMI-SDS -1.09 ± .34) from a Northern Finnish Birth Cohort 1986 [20] (n=5,291; called ‘Finnish’ in Table S3) were also accessed. The genotyping of SNPs in these samples was done using the Applied Biosystems SNPlex™ technology ([http://www.appliedbiosystems.com](http://www.appliedbiosystems.com/)) based on the Oligonucleotide Ligation Assay combined with multiplex PCR target amplification. Quality control criteria: individual SNP call rates ≥ 95%, Hardy-Weinberg equilibrium in the control samples (p ≥ .01).

- - 1. Family-based association studies with extremely obese children and adolescent case-control studies of European origin

715 nuclear families comprising 981 (534 females) extremely obese children and adolescents with an age and gender specific BMI ≥ 89th BMI percentile (78% had a BMI ≥ 97th BMI percentile; mean age 13.80 ± 3.83 years; mean BMI 30.91 ± 6.02 kg/m²; mean BMI-SDS 3.87 ± 2.01) and both of their biological parents were recruited in hospitals specialized for the inpatient treatment for extreme obesity. The index patients were independent of the German GWAS cases. The ascertainment strategy was previously described in detail [21]. Written informed consent was given by all participants and in case of minors by their parents. The study was approved by the Ethics Committees of the Universities of Marburg and Essen and conducted in accordance with the guidelines of *The Declaration of Helsinki.* Genotyping was carried out by matrix-assisted desorption/ionization time-of-flight mass spectrometry (MALDI-TOF MS; Sequenom iPLEX^TM^) analysis of allele-dependent primer extension products as described elsewhere [22,23]. The genotyping was performed at the Helmholtz Zentrum, München. Genotyping quality control criteria we calculated individual SNP call rates and Hardy-Weinberg equilibrium (exact two-sided p-values) in the founders. Note that for the markers rs2968959, rs2968941, and rs538656 call rates were ≤ 90% and that for the markers rs2011946, rs3898382, rs12706717, and rs918657 the Hardy-Weinberg equilibrium exact two-sided p-values < .01. As for all markers expect for rs2011946 proxy markers in the same region were available, it was decided to report these results nevertheless. In the SHIP sample (see below) the proxies rs11693502 and rs925966 for rs2011946 lead to p-values of .27 and .18 in the linear regression (additive genetic model) for BMI adjusted for age and sex and the direction of the effect was different than expected from the GWAS.

- - 1. Marker selection for GENERALIZATION

We selected markers for the GENERALIZATION after the DISCOVERY steps based on the following criteria:

- the association had to be directionally consistent (i.e. we observed the same obesity risk effect allele as in our GWAS meta-analysis)
- within each region the minimum combined p-value had to be ≤ 5x10^-4^
- for each SNP this meant:
  - directionally consistent obesity risk effect alleles in the DISCOVERY step
  - two-sided p-value in one of the case-control comparisons had to be ≤ .05
  - two-sided p-value of the family-based association test had to be ≤ .15

We used the software tools SNAP [24] and CandiSNPer [25] to display candidate gene regions.

- - 1. Power considerations for the Follow-up

Power considerations using QUANTO 1.2.3 (<http://hydra.usc.edu/gxe>) revealed that a sample of 1,181 cases and 1,960 controls and a sample of 715 nuclear families with one obese offspring has a combined comparison-wise power ≥ .8 to detect a log-additive OR ≥ 1.35 for minor allele frequencies (MAFs) of 10 % (α=.002; one-sided; corrected for 21 regions of interest; rare disease assumption). 10% was the lowest MAF found in all SNPs followed-up from GWAS meta-analysis for early-onset obesity.

# GENERALIZATION

## Children and adolescents of European origin

- - 1. Obesity sample: Datteln Paediatric Obesity sample (T. Reinehr)

The Datteln Paediatric Obesity sample included overweight or obese children and adolescents which were either recruited at the “Vestische Kinderklinik Datteln”, University of Witten/Herdecke, Germany. Some of the latter participated in a standardised lifestyle intervention program “Obeldicks” [26]. The ascertainment strategy was previously described in detail [27]. Briefly, the investigated overweight and obese children had no endocrine or syndromal disorders and came to our outpatient centre specialized in pediatric obesity and endocrinology from 1999 onwards. All individuals were of European descent. Written informed consent was given by all participants and in the case of minors by their parents. The study was approved by the ethics committees of the Universities of Witten/Herdecke and carried out according to *The Declaration of Helsinki*. Here we report data for 711 (399 girls) children and adolescents (all: mean BMI 27.49 ± 4.97, mean BMI-SDS 2.37 ± .54, mean age 10.71 ± 2.73; girls: mean BMI 27.44 ± 5.26, mean BMI-SDS 2.36 ± .57, mean age 10.76 ± 2.84; boys: mean BMI 27.55 ± 4.59, mean BMI-SDS 4.59 ± 2.38, mean age 10.63 ± 2.60) which were used as cases in the case-control analysis of the GENERALIZATION step.

Genotyping was carried out by MALDI-TOF MS at the Helmholtz Zentrum, München, and genotyping quality control criteria were: individual SNP call rates ≥ 99%, and Hardy-Weinberg equilibrium (p ≥ .15) even though this should not be used to exclude markers in selected samples.

- - 1. Population-based samples: GINI/LISA (J. Heinrich, P. Rzehak, D. Berdel, M. Borte) and Berlin School Girls sample (H. Krude, H. Biebermann)

For the birth cohorts GINI /LISA [28] 10,000 random population-based newborns were recruited in the years 1995-1999 in four areas spread across Germany. The cohort has been followed-up for almost 10 years; here we report cross-sectional data from the 6 year follow up. For GINI 1,694 (829 girls) children were genotyped (GINI all: mean BMI 15.40 ± 1.35, mean BMI-SDS -.08 ± .88, mean age 5.22 ± .17; GINI girls: mean BMI 15.42 ± 1.37, mean BMI-SDS .01 ± .98, mean age 12.68 ± 1.19; GINI boys: mean BMI 15.38 ± 1.34, mean BMI-SDS -.12 ± .90, mean age 5.22 ± .17). For LISA 952 (434 girls) children were genotyped (all: mean BMI 15.29 ± 1.38, mean BMI-SDS -.16 ± .90, mean age 5.21 ± .16; LISA girls: mean BMI 15.20 ± 1.40, mean BMI-SDS -.18 ± .87, mean age 5.21 ± .17; LISA boys: mean BMI 15.37 ± 1.37, mean BMI-SDS -.13 ± .93, mean age 5.20 ± .16).

Another population-based cross-sectional sample of school girls was recruited from 68 Berlin schools in 2005 and 2006. DNA was extracted by an adopted method based on chewing gums. From this population of school girls, we report data for 879 girls of European origin (mean BMI 19.13 ± 3.03, mean BMI-SDS .01 ± .98, mean age 12.68 ± 1.19).

Genotyping was carried out by MALDI-TOF MS at the Helmholtz Zentrum, München, and genotyping quality control criteria were: individual SNP call rates ≥ 95%, and Hardy-Weinberg equilibrium (p ≥.05) except for rs11127485 (p=.003) where also the call rate was only 94%. Nonetheless based on the results of the other samples, it was decided to include the data on this marker.

## Adults of European origin

- - 1. Obesity sample: Marburg Adult Obesity sample (W. Rief)

The Marburg adult obesity sample [29] is a sample of German obese adults ascertained in the city and region of Marburg (Hessia, Germany) by general practitioners. The sole inclusion criterion was a BMI ≥ 30 kg/m^2^. Written informed consent was given by all participants. The study was approved by the Ethics Committees of the Universities of Marburg and carried out according to *The Declaration of Helsinki*. Here we report data for 988 (626 females) adults (all: mean BMI 36.02 ± 5.38, mean age 46.31 ± 14.76; females: mean BMI 36.54 ± 5.66, mean age 46.15 ± 15.05; males: mean BMI 35.14 ± 4.73, mean age 46.59 ± 14.26). Genotyping was carried out by MALDI-TOF MS at the Helmholtz Zentrum, München, and genotyping quality control criteria were: individual SNP call rates ≥ 95%, and Hardy-Weinberg equilibrium (p ≥ .15) except for rs1558902 (p=.002) which might indicate the association to *FTO*.

- - 1. Population-based sample: The EPIC-Potsdam Study (H. Boeing, E. Fisher)

This EPIC (*‘European Prospective Investigation into Cancer and Nutrition’*) study is a longitudinal, multicenter study involving approximately 0.5 million participants recruited in ten European countries. In Potsdam [30], 27,548 study participants mainly in the age range 35 to 65 years underwent baseline examination between 1994 and 1998. The baseline examination included anthropometric and blood pressure measurements, blood sampling, a self-administered validated food-frequency questionnaire, and a personal interview on lifestyle habits and medical history. Informed consent was obtained from all study participants, and approval was given by the Ethical Committee of the State of Brandenburg, Germany. Here, we selected a random subsample (5,000 subjects) from the entire cohort (all: mean BMI 26.3 ± 4.4, mean age 49.8 ± 8.9; females: mean BMI 25.9 ± 4.8, mean age 48.6 ± 9.2; males: mean BMI 26.9 ± 3.6, mean age 51.8 ± 8.1). Genotyping was done by KBioscience (Hoddesdon, UK) and genotyping quality control criteria were: individual SNP call rates ≥ 95%, Hardy-Weinberg equilibrium (exact two-sided p-values ≥ .15).

- - 1. Population-based sample: KORA (H.E. Wichmann, T. Illig, H. Grallert)

KORA-Augsburg (Kooperative Gesundheitsforschung im Raum Augsburg; ‘*Cooperative Health Research in the Region of Augsburg*’; <http://www.helmholtz-muenchen.de/kora/> ) is an epidemiological study group including a total of about 18,000 men and women aged 25 to 74 years at recruitment, for all of which DNA samples are available. The KORA surveys have been described in detail previously [31]. All KORA participants have a German passport and are of European origin.

Here we report data for 12,002 (5,971 females) adults of the S2-4 surveys (all: mean BMI 26.97 ± 4.49, mean age 49.39 ± 13.94; females: mean BMI 26.62 ± 5.07, mean age 49.05 ± 13.77; males: mean BMI 27.31 ± 3.80, mean age 49.72 ± 14.09).

Genotyping was carried out by MALDI-TOF MS at the Helmholtz Zentrum, München, and genotyping quality control criteria were: individual SNP call rates ≥ 95 % (except for rs1127485 where 6.5% were missing), and Hardy-Weinberg equilibrium was p ≥ .01 for all SNPs except for rs516175 (p=.0006) and rs1558902 (p=.004). For all three markers it was nevertheless decided to report the results as their results were either consistent with the results for proxy or with the results in the other population-based samples.

- - 1. Population-based sample: SHIP (D. Rosskopf, C. Rimmbach)

The *Study of Health in Pomerania* (SHIP [32]; <http://www.medizin.uni-greifswald.de/cm/fv/ship.html>) is a cross-sectional population-based general health survey in Northeast Germany and comprises a total of 4,310 individuals aged 20 to 79 years at recruitment. The design of the study is similar to that of the KORA sample (3.2.3). Here we report Affymetrix Genome-Wide Human SNP Array 6.0 data for 4,310 (2,192 females) adults (all: mean BMI 27.28 ± 4.77, mean age 49.79 ± 16.40) at the baseline examination. For rs1558902, rs9935401, and rs17700144 we only report marker data on proxies which had an estimated r^2^=1 with the source marker [24]. Quality control criteria in the GWAS were: individual SNP call rates ≥ 95%, Hardy-Weinberg equilibrium in the reported SNPs (p > .5).

- - 1. Population-based sample: The Heinz-Nixdorf Recall Study (K.H. Jöckel, R. Erbel, S. Moebus)

The Heinz Nixdorf Recall cohort [33] (<http://www.recall-studie.uni-essen.de/>) comprises 4,814 individuals aged 45-74 years at baseline (2000-2003) and includes 4 years of questionnaire-based mortality and morbidity follow-up (cardiovascular disease, cancer); a 2nd examination is currently being performed in the 5th year (2006-2008). Here we report data for 4,646 (2,317 females) adults (all: mean BMI 27.72 ± 5.35, mean age 59.61 ± 7.81; females: mean BMI 27.43 ± 5.88, mean age 59.58 ± 7.82; males: mean BMI 28.19 ± 3.94, mean age 59.65 ± 7.79). Genotyping was carried out by the MALDI-TOF mass spectrometry-based iPLEX Gold assay at the Department of Genomics, Life & Brain Center, Bonn and genotyping quality control criteria were: individual SNP call rates ≥ 99% (except for rs1558902 and rs9935401 for which 5-10% genotypes were missing – as data for the proxy rs8050136, which had a call rate ≥ 99%, were similar it was decided not to discharge these SNPs) and Hardy-Weinberg equilibrium (p ≥ .01).

References for the Supporting Information

1. Visvikis-Siest S, Siest G (2008) The STANISLAS Cohort: a 10-year follow-up of supposed healthy families. Gene-environment interactions, reference values and evaluation of biomarkers in prevention of cardiovascular diseases. Clin Chem Lab Med 46: 733-747.

2. Jaquet D, Collin D, Levy-Marchal C, Czernichow P (2004) Adult height distribution in subjects born small for gestational age. Horm Res 62: 92-96.

3. Meyre D, Delplanque J, Chevre JC, Lecoeur C, Lobbens S, et al. (2009) Genome-wide association study for early-onset and morbid adult obesity identifies three new risk loci in European populations. Nat Genet 41: 157-159.

4. Hinney A, Nguyen TT, Scherag A, Friedel S, Brönner G, et al. (2007) Genome wide association (GWA) study for early onset extreme obesity supports the role of fat mass and obesity associated gene (FTO) variants. PLoS ONE 2: e1361.

5. Korn JM, Kuruvilla FG, McCarroll SA, Wysoker A, Nemesh J, et al. (2008) Integrated genotype calling and association analysis of SNPs, common copy number polymorphisms and rare CNVs. Nat Genet 40: 1253-1260.

6. Purcell S, Neale B, Todd-Brown K, Thomas L, Ferreira MA, et al. (2007) PLINK: a tool set for whole-genome association and population-based linkage analyses. Am J Hum Genet 81: 559-575.

7. Nothnagel M, Ellinghaus D, Schreiber S, Krawczak M, Franke A (2009) A comprehensive evaluation of SNP genotype imputation. Hum Genet 125: 163-171.

8. Marchini J, Howie B, Myers S, McVean G, Donnelly P (2007) A new multipoint method for genome-wide association studies by imputation of genotypes. Nat Genet 39: 906-913.

9. Purcell S, Cherny SS, Sham PC (2003) Genetic Power Calculator: design of linkage and association genetic mapping studies of complex traits. Bioinformatics 19: 149-150.

10. Frayling TM, Timpson NJ, Weedon MN, Zeggini E, Freathy RM, et al. (2007) A common variant in the FTO gene is associated with body mass index and predisposes to childhood and adult obesity. Science 316: 889-894.

11. Willer CJ, Speliotes EK, Loos RJ, Li S, Lindgren CM, et al. (2009) Six new loci associated with body mass index highlight a neuronal influence on body weight regulation. Nat Genet 41: 25-34.

12. Thorleifsson G, Walters GB, Gudbjartsson DF, Steinthorsdottir V, Sulem P, et al. (2009) Genome-wide association yields new sequence variants at seven loci that associate with measures of obesity. Nat Genet 41: 18-24.

13. Rankinen T, Zuberi A, Chagnon YC, Weisnagel SJ, Argyropoulos G, et al. (2006) The human obesity gene map: the 2005 update. Obesity (Silver Spring) 14: 529-644.

14. Scuteri A, Sanna S, Chen WM, Uda M, Albai G, et al. (2007) Genome-wide association scan shows genetic variants in the FTO gene are associated with obesity-related traits. PLoS Genet 3: e115.

15. Geller F, Reichwald K, Dempfle A, Illig T, Vollmert C, et al. (2004) Melanocortin-4 receptor gene variant I103 is negatively associated with obesity. Am J Hum Genet 74: 572-581.

16. Stutzmann F, Vatin V, Cauchi S, Morandi A, Jouret B, et al. (2007) Non-synonymous polymorphisms in melanocortin-4 receptor protect against obesity: the two facets of a Janus obesity gene. Hum Mol Genet 16: 1837-1844.

17. Benzinou M, Creemers JW, Choquet H, Lobbens S, Dina C, et al. (2008) Common nonsynonymous variants in PCSK1 confer risk of obesity. Nat Genet 40: 943-945.

18. Heude B, Kettaneh A, Rakotovao R, Bresson JL, Borys JM, et al. (2005) Anthropometric relationships between parents and children throughout childhood: the Fleurbaix-Laventie Ville Sante Study. Int J Obes (Lond) 29: 1222-1229.

19. Gelbrich G, Bluher S, Reich A, Muller G, Kiess W (2008) Prevalence of obesity and elevated blood pressure as well as onset of puberty in German school children attending different educational tracks. Horm Res 70: 340-348.

20. Jarvelin MR, Elliott P, Kleinschmidt I, Martuzzi M, Grundy C, et al. (1997) Ecological and individual predictors of birthweight in a northern Finland birth cohort 1986. Paediatr Perinat Epidemiol 11: 298-312.

21. Hinney A, Lentes KU, Rosenkranz K, Barth N, Roth H, et al. (1997) Beta 3-adrenergic-receptor allele distributions in children, adolescents and young adults with obesity, underweight or anorexia nervosa. Int J Obes Relat Metab Disord 21: 224-230.

22. Vollmert C, Windl O, Xiang W, Rosenberger A, Zerr I, et al. (2006) Significant association of a M129V independent polymorphism in the 5' UTR of the PRNP gene with sporadic Creutzfeldt-Jakob disease in a large German case-control study. J Med Genet 43: e53.

23. Ye S, Dhillon S, Ke X, Collins AR, Day IN (2001) An efficient procedure for genotyping single nucleotide polymorphisms. Nucleic Acids Res 29: E88.

24. Johnson AD, Handsaker RE, Pulit SL, Nizzari MM, O'Donnell CJ, et al. (2008) SNAP: a web-based tool for identification and annotation of proxy SNPs using HapMap. Bioinformatics 24: 2938-2939.

25. Schmitt AO, Assmus J, Bortfeldt RH, Brockmann GA (2010) CandiSNPer: a web-tool for the identification of candidate SNPs for causal variants. Bioinformatics

26. Reinehr T, Temmesfeld M, Kersting M, de SG, Toschke AM (2007) Four-year follow-up of children and adolescents participating in an obesity intervention program. Int J Obes (Lond) 31: 1074-1077.

27. Reinehr T, Hinney A, de SG, Austrup F, Hebebrand J, et al. (2007) Definable somatic disorders in overweight children and adolescents. J Pediatr 150: 618-22, 622.

28. Chen CM, Morgenstern V, Bischof W, Herbarth O, Borte M, et al. (2008) Dog ownership and contact during childhood and later allergy development. Eur Respir J 31: 963-973.

29. Hinney A, Bettecken T, Tarnow P, Brumm H, Reichwald K, et al. (2006) Prevalence, spectrum, and functional characterization of melanocortin-4 receptor gene mutations in a representative population-based sample and obese adults from Germany. J Clin Endocrinol Metab 91: 1761-1769.

30. Boeing H, Wahrendorf J, Becker N (1999) EPIC-Germany--A source for studies into diet and risk of chronic diseases. European Investigation into Cancer and Nutrition. Ann Nutr Metab 43: 195-204.

31. Holle R, Happich M, Lowel H, Wichmann HE (2005) KORA--a research platform for population based health research. Gesundheitswesen 67 Suppl 1: S19-S25.

32. Völzke H, Wallaschofski H, Wolff B, Berger K, John U, et al. (2006) Thyroid function and serum ferritin levels: the study of health in Pomerania. Thyroid 16: 681-686.

33. Schmermund A, Baumgart D, Sack S, Möhlenkamp S, Grönemeyer D, et al. (2000) Assessment of coronary calcification by electron-beam computed tomography in symptomatic patients with normal, abnormal or equivocal exercise stress test. Eur Heart J 21: 1674-1682.
